# Perturbed actin cap as a new personalized biomarker in primary fibroblasts of Huntington’s disease patients

**DOI:** 10.3389/fcell.2023.1013721

**Published:** 2023-01-18

**Authors:** Saja Gharaba, Omri Paz, Lea Feld, Anastasia Abashidze, Maydan Weinrab, Noam Muchtar, Adam Baransi, Aviv Shalem, Uri Sprecher, Lior Wolf, Haguy Wolfenson, Miguel Weil

**Affiliations:** ^1^ Laboratory for Personalized Medicine and Neurodegenerative Diseases, The Shmunis School of Biomedicine and Cancer Research, The George S. Wise Faculty for Life Sciences, Sagol School of Neurosciences, Tel Aviv University, Tel Aviv, Israel; ^2^ Department of Genetics and Developmental Biology, The Rappaport Faculty of Medicine and Research Institute, Technion—Israel Institute of Technology, Haifa, Israel; ^3^ The Blavatnik Center for Drug Discovery, Tel Aviv University, Tel Aviv, Israel; ^4^ The Blavatnik School of Computer Sciences, Tel Aviv University, Tel Aviv, Israel; ^5^ School of Electrical Engineering, Faculty of Engineering, Tel Aviv University, Tel Aviv, Israel

**Keywords:** huntington’s disease, actin cap, nuclear morphology, primary skin fibroblast, personalized drug screening, image-based high content analysis, disease marker

## Abstract

Primary fibroblasts from patient’s skin biopsies are directly isolated without any alteration in the genome, retaining in culture conditions their endogenous cellular characteristics and biochemical properties. The aim of this study was to identify a distinctive cell phenotype for potential drug evaluation in fibroblasts from Huntington’s Disease (HD) patients, using image-based high content analysis. We show that HD fibroblasts have a distinctive nuclear morphology associated with a nuclear actin cap deficiency. This in turn affects cell motility in a similar manner to fibroblasts from Hutchinson-Gilford progeria syndrome (HGPS) patients used as known actin cap deficient cells. Moreover, treatment of the HD cells with either Latrunculin B, used to disrupt actin cap formation, or the antioxidant agent Mitoquinone, used to improve mitochondrial activity, show expected opposite effects on actin cap associated morphological features and cell motility. Deep data analysis allows strong cluster classification within HD cells according to patients’ disease severity score which is distinct from HGPS and matching controls supporting that actin cap is a biomarker in HD patients’ cells correlated with HD severity status that could be modulated by pharmacological agents as tool for personalized drug evaluation.

## Introduction

Huntington’s disease (HD) is a fatal rare inherited disorder with a broad impact on a person’s functional abilities characterized by unwanted choreatic movements, behavioral and psychiatric disturbances, and dementia (Kirkwood et al., 2001; Walker, 2007). HD is an autosomal dominant disease caused by elongated CAG repeats on the short arm of chromosome 4p16.3 in the huntingtin gene (HTT). Normally, the CAG segment is repeated 10–35 times within the gene, but in people with HD, the CAG segment is repeated 36 to more than 120 times, causing a mutated translated protein with long Poly glutamine (Poly-Q) repeats called mutant Huntingtin protein (mHtt). The Huntingtin protein is expressed in all human and mammalian cells (Strong et al., 1993; Dé Ric Saudou and Humbert, 2016). The role of the Htt protein in humans is unclear. It interacts with over 100 other proteins that are involved in a number of biological functions like transcription, cell signaling, and intracellular transport (Harjes and Wanker, 2003). Although the mutated protein function is poorly understood, it is toxic to certain cell types due to Poly-Q mediated protein aggregation, particularly in the brain (Moily et al., 2017). Notably, it has become clear from different studies that HD is not only a brain disorder, but a multisystem disease that affects numerous cell types (Sathasivam et al., 1999; Mielcarek, 2015). It is therefore appealing to use easily accessible cells from patients for phenotype characterization and *in vitro* studies.

Human primary skin fibroblast cells from patient’s skin biopsies were used previously as a model to study different neurodegenerative diseases (Connolly, 1998; Huang et al., 2006), including HD (Marchina et al., 2014; Gardiner et al., 2018). These cells are directly isolated from the patient’s tissue without any alteration in the genome (Alge et al., 2006), retaining in culture conditions their endogenous cellular characteristics and biochemical properties, as well as their cellular proliferation capacity for several passages. This allows performing multiple experiments in a reproducible manner from a single patient’s sample and identify distinctive cellular phenotypes and potential disease biomarkers also expressed in more disease-relevant tissues *in vivo*, as previously described for Amyotrophic Lateral Sclerosis patients’ bone marrow mesenchymal stem cells (Wald-Altman et al., 2017). Here we adopted image-based high content analysis (HCA) cell phenotyping (Solmesky and Weil, 2014) as an unbiased approach for screening of robust and distinctive subcellular morphological features in primary skin fibroblasts of HD patients. We found altered nuclear morphology as a significant phenotypic feature of HD cells. Detailed confocal and deep morphometric image analyses of the nuclei, combined with cell migration assays, revealed that this abnormal morphology is due to a nuclear actin cap deficiency in HD patients’ fibroblasts. The nuclear actin cap is an organized apical dome that covers the nucleus and is composed of thick, parallel, and highly contractile actin and myosin filament bundles (Khatau et al., 2009). Actin cap formation is functionally related to cell migration, intranuclear shaping, chromosomal organization (Kim et al., 2014), and cellular mechanoregulation (Kim et al., 2013). The actin cap is present in a wide range of adherent eukaryotic cells, but it is disrupted in several human diseases such as cancer (Zink et al., 2004) and muscular dystrophy (Booth-Gauthier et al., 2013). It is a distinctive biomarker in Hutchinson–Gilford syndrome (HGPS), the accelerated nuclear laminopathy aging syndrome also known as progeria, caused by a dominant mutation in LMNA gene affecting Lamin A/C levels (Khatau et al., 2009; Kim et al., 2017). Our results demonstrate that actin cap deficiency is a robust biomarker in HD primary skin fibroblast cells that could be used as a powerful functional measure in drug screening assays as well as for future personalized drug test validations for treatment of HD patients.

## Materials and methods

Most of the reagents used in this study (otherwise stated) were purchased from Thermo Fischer Scientific, United States of America.

### Primary skin fibroblasts samples and cell culture handling

Primary skin fibroblasts (see Table 1, Table 2) used in this study were obtained from different cell and tissue repositories as mentioned by the ethical declaration of this manuscript. The cells from 28 HD patients and 19 matched healthy controls (age 23–68), and from 6 HGPS and 4 young healthy controls (age 1–11) were grown in culture media DMEM supplemented with 1% MEM Sodium Pyruvate, 1% PSA (Biological industries, Israel), 10% heat inactivated FBS, and 1% NEAA in polystyrene plastic 75-cm2 culture flasks (Corning, NY) at 37°C with 5% CO2. Cell passages and expansion of human skin fibroblasts were performed when cells were at 80%–100% confluency. Cells passaging and re-plating was accomplished using .25% Trypsin-EDTA (Biological industries, Israel) for 2 min, followed by addition of twice the volume of a complete culture media to neutralize the enzyme. Cells were subsequently centrifuged at 1200 rpm for 5 min before their pellets being resuspended in 1 ml medium for cell counting using TC10 automated cell counter (BioRad United State) before re-plating. All experiments were performed between passages 7–15.

**TABLE 1:** List of skin fibroblasts samples of HD individuals.

HD individual	Age	Gender	CAG Repeats	CAP Score- Fneur	Disease Severity	Figure Number
GM02165	57 YR	Male	46^a^	140.5239	Severe	4
GM05031	60 YR	Male	45^a^	138.6749	Severe	4
GM04476	57 YR	Male	45^b^	131.7411	Severe	1,2,3,4
GM04200	53 YR	Male	46^a^	130.6626	Severe	4
GM00305	56 YR	Female	45^a^	129.4299	Severe	4
GM04285	40 YR	Male	51^b^	129.4299	Severe	4
GM06274	56 YR	Female	45^a^	129.4299	Severe	1,4
GM04807	41 YR	Male	50^b^	126.3482	Severe	4
GM04287	43 YR	Male	49^b^	125.886	Severe	1,2,4,5
GM02147	55 YR	Male	44^a^	118.6441	Severe	4
GM04691	31 YR	Male	54^b^	114.6379	Severe	4
GM04687	37 YR	Female	50^b^	114.0216	Severe	4
GM04709	38 YR	Female	49^b^	111.2481	Mild	1,2,3,4,5
GM04715	40 YR	Male	48^b^	110.9399	Mild	3,4,5
GM04887	48 YR	Female	45^b^	110.9399	Mild	1,2,3,4
GM04196	51 YR	Female	44^a^	110.0154	Mild	1,2,4
GM04767	43 YR	Female	46^b^	106.0092	Mild	4
GM04849	28 YR	Female	54^a^	103.5439	Mild	4
GM04212	50 YR	Female	43^a^	100.1541	Mild	1,2,4,5
GM04819	48 YR	Male	43^b^	96.14792	Mild	1,2,4,5
GM04799	47 YR	Male	43^b^	94.14484	Mild	1,2,4
GM04721	37 YR	Female	46^b^	91.21726	Mild	1,2,3,4
GM04719	39 YR	Female	44^b^	84.12943	Premanifest	1,2,4
GM04847	31 YR	Male	46^b^	76.42527	Premanifest	4
GM04693	33 YR	Male	45^b^	76.27119	Premanifest	4
GM04717	44 YR	Female	41^b^	74.57627	Premanifest	1,2,3,4
GM04689	30 YR	Female	45^b^	69.33744	Premanifest	4
GM04837	23 YR	Male	47^b^	60.24653	Premanifest	4

^a^ CAG repeat number of the HD sample was obtained using AmplideX® PCR/CE HTT Kit following the manufacturers protocol guidelines.

^b^ CAG repeat number of the HD sample was provided by Coriell Institute Cell Repository.

**TABLE 2:** List of skin fibroblasts control samples (Adult HC, Young HC, and HGPS).

HC individual	Age	Gender	Figure Number	HGPS individuals	Age	Gender	Figure Number
0205C	68 YR	Female	1,2,4	HGADFN367	3 YR 0 mos	Female	2,4
0495C	53 YR	Female	4	HGADFN188	2 YR 3 mos	Female	2,4
0951C	61 YR	Female	4	HGADFN169	8 YR 6 mos	Male	2,4,5
0025C	48 YR	Male	1,2,3,4	HGADFN122	5 YR `0 mos	Female	2,4,5
0553C	49 YR	Male	1,2,4	HGADFN127	3 YR 9 mos	Female	2,4
0795C	50 YR	Male	1,2,4	HGADFN271	1 YR 3 mos	Male	2,4
0971C	56 YR	Male	4				
1170C	43 YR	Male	1,2,3,4,5	**Young HC individuals**			
0143C	42 YR	Male	1,2,4,5	GM2036	11 YR	Female	2,4
0981C	50 YR	Male	1,2,4	07525C	4 YR	Male	2,4
0561C	48 YR	Male	1,2,4	0015C	1 YR 6 mos	Male	2,4
GM00726	26 YR	Female	4	0044C	2 YR	Male	2,4
GM01650	37 YR	Female	4				
GM01653	37 YR	Male	4				
1016C	62 YR	Male	4				
0848C	48 YR	Female	1,2,3,4,5				
0730C	51 YR	Male	4				
0633C	50 YR	Male	4				
0579C	40 YR	Male	1,2,4				
0233C	61 YR	Female	1,2,4				

### Image based cell HCA live phenotyping experiments

For live image microscopy experiments, 1500 cells in 100 μL of complete medium per well were automatically plated in 96-well plates (Grenier, Austria) using Tecan Freedom EVO 200 robot equipped with a 96 MultiChannel Arm (MCA). The plates accommodated serially and in columns, alternating 5 cell samples from each group HD and HC. All steps of the seeding, washing, staining, replacing media protocols were programmed for running automatically with optimized pipetting parameters for each task. After 24 h incubation at 37°C and 5% CO2, culture media was removed and replaced, after one wash with DPBS, with fluorescent dyes mix diluted in HBSS. After 30 min incubation at 37°C, and 5% CO2 the plate was transferred to the IN Cell Analyzer 2200 (GE Healthcare) for image acquisition under cell culture environmental conditions. The fluorescent vital dyes mix used contain Hoechst 33,342 (Merck-Sigma, Unite State) 1:10,000, CellTrace™ Calcein Green AM (Invitrogen, Unite State) 1:5000, mitochondria TMRE red (Invitrogen, Unite State) 1:2500 and MitoTracker™ Deep Red (Invitrogen, Unite State) 1:5000. Twenty images per fluorescent channel (fixed spacing fields) for each well were acquired in four different channels in less than 60 min. All images under a 20x magnification were taken using the same acquisition protocol with constant exposures for each fluorescent channel for each of the four fluorescent dyes used to stain the cells. Image acquisition has been done in a horizontal serpentine pattern. The multiple cell images were subsequently segmented and high content analyzed using IN Cell Developer software (GE Healthcare).

### Actin F and nuclear staining assay for fluorescent microscopy

Cells from HC and HD samples were seeded and culture in 96 well plates as described above, then fixed in 4% v/v Paraformaldehyde (Electron Microscopy Sciences, Unite State) in PBS for 10 min, and washed 3 times with DPBS. Cells were then permeabilized with .1% Triton X-100 (Merk-Sigma, Unite State) in PBS for 10 min at room temperature. Then Blocking solution 5% w/v BSA (Chem-Impex, Unite State) in TBST was added to the cells for 1 h. After 1 h. After 1 h, the sample was washed 3 times with .05% Triton X-100 for 5 min. After the removal of the 5% BSA solution.

The cells were washed with PBS and the nuclei and actin filaments were stained at room temperature for 1 h in the dark with a mix of 30 μL Hoechst 33,342 1:10,000 and Phalloidin (Merk-Sigma, Unite State) 1:400 in 5% BSA (in TBST) respectively. The stained cells were washed three times with DPBS and image acquisition and analysis of the plate was performed similarly as described above.

### Confocal microscopy, image and data analysis

Confocal microscopy of actin and nuclei labeled cells for actin cap validation experiments was performed under a Leica SP8 LIGHTNING (Leica Microsystems, Germany) confocal microscope with a 40x 1.2 N.A objective using 561 nm and 405 nm laser wavelengths for actin fibers and nucleus, respectively. The analysis of the actin cap fibers at the at the apical and basal focal planes was done using the LAS X software (Leica Microsystems, Germany). To determine different morphological clusters of actin cap in the HD skin fibroblast confocal microscopy analysis was performed using a Zeiss LSM800 confocal microscope with a 63x 1.4NA objective and Airyscan module. A region of interest of 40.6 µm by 40.6 µm and scan zoom of 2.5 was acquired using 561 nm and 405 nm laser wavelengths for actin fibers and nucleus, respectively. Images were processed using the Airyscan algorithm in the ZEN blue software (Zeiss). Actin cap fibers were identified using Trainable Weka segmentation plugin for FIJI (Arganda-Carreras et al., 2017), and thresholding was used to define the nucleus region. Fiber orientation analysis was done using the Ridge Detection and Orientation J plugins for FIJI.

### Cell migration time lapse microscopy assay

The primary fibroblasts were plated in a 96-well plate at low density (800 cells/well) and then incubated overnight at 37°C and 5% CO2. Time lapse microscopy imaging of these plated cells was performed using an IN Cell Analyzer 2200 equipped with a long working distance 4× objective, phase contrast capability and controlled environmental chamber. The image acquisition frequency was set every 10 min for 21 h for 30 wells. The analysis of the time lapse images was performed by CellTracker program using semi-automated cell tracking function which allows a quantitative determination of cell motion parameters, including cell total travel length, net displacement and velocity. The cells paths were plotted and analyzed through The Chemotaxis and Migration Tool (ImageJ plug-in) to create the sun plots shown in the results.

### Custom image analysis tool for actin cap like morphological features

The image analysis tool was fully programmed and validated in our lab using python 3.7 programming language https://gitlab.com/maydanw/CellDoctor.

The image analysis script can be found under: https://gitlab.com/maydanw/CellDoctor/-/blob/master/Notebooks/Run_Image_Analysis.ipynb. The image analysis script is divided into two parts. The first part is for linking between the image channel specification (e.g., DAPI - DAPI) to the logical channel (e.g., NucliChannel). The second part is for extracting different morphological features of a particular object (in DataExtractors). The overall process of the image analysis tool is described in the “Protocol” section. The images were mostly processed by different functions of the OpenCV library.

The first step in image pre-processing is applying image binarization using binary thresholding which turns the raw image into a grayscale. Followed by noise reduction using image thresholding. Various techniques for object detection and segmentation were used according to the label and to the object morphology. For example, on the nuclei image a Simple Binary Thresholding was applied where for every pixel the same threshold value is applied. If the pixel value is smaller than the threshold, it is set to 0, otherwise it is set to a maximum value = 255. On the Binary Thresholding different morphological operations like Erosion, Dilation, Opening and Closing etc. was applied through the Morphological Transformation which is one of the Image Processing applications in OpenCV.

Microscopy images of skin fibroblast cells were processed through the tool, extracting the following actin cap like parameters: The nucleus circularity.

The standard deviation of actin fibres’ slope in each cell–slopes of all actin fibers were calculated per cell followed by a calculation of the standard deviation of the slopes per cell.

$$Circularity=\left(4\;*\;pi\;*\;Area\right)\;/\;Perimeter^{\wedge }2$$

$$slope=\frac{y_{1}-y_{2}}{x_{1}-x_{2}}\;STD=\sqrt{{\left(\frac{1}{N}\overset{N}{\underset{i=1}{\sum }}\left(slope_{i}-\bar{slope}\right.\right)}^{2}}$$

### Statistical analysis

The raw data obtained from the image analysis in this study was further statistically analyzed using the imaging assay development notebook script at https://github.com/disc04/simplydrug site, found under HTS notebook, under imaging-based assays development script being adapted to our data. For checking if the two groups were significantly different from each other, the Mann-Whitney U test was used on the medians of different parameters comparing between two groups using GraphPad 8, and the results were presented in box plots using Tableau 2020.3. For graph generations. The stars presented on the different graphs indicate different significance levels (*) *p*-value <.05 (**) *p*-value <.01 (***) *p*-value <.001.

### Data sharing

The data that support the findings of this study are available on request from the corresponding author. The data are not publicly available due to privacy or ethical restrictions.

## Results

### Primary skin fibroblasts from HD patients display distinct morphological features

**FIGURE 1:**
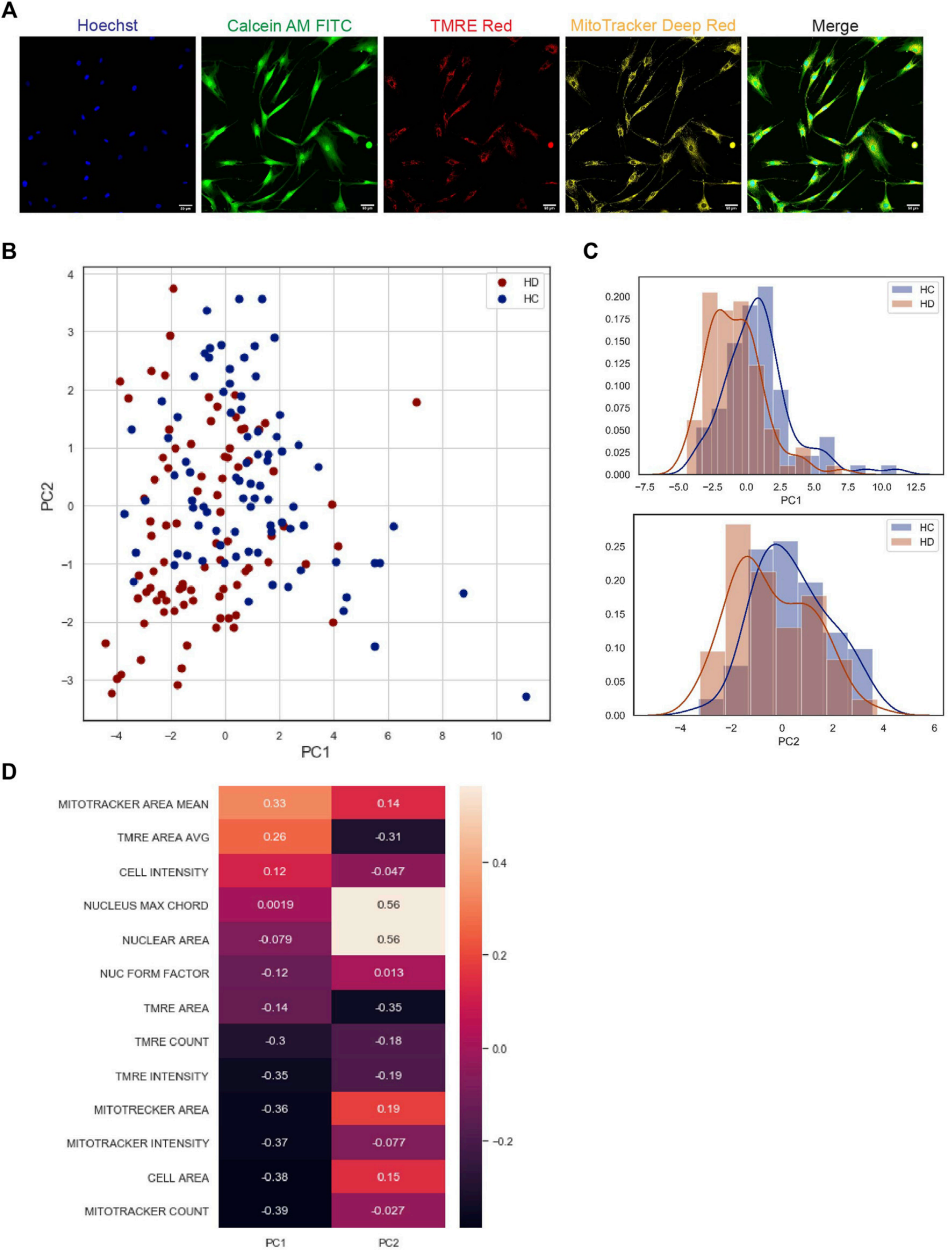
High content image based analysis of primary skin fibroblast samples of HD patients compared to HC **(A)** Representative images of skin fibroblast cells stained with 4 different fluorescent vital dyes (Hoechst 22,342 to label the cell nuclei, Calcein AM to label cytoplasm of living cells, TMRE to label functional mitochondria, and Mitotracker far-red to label mitochondria). The images were acquired by 20x magnification using INCell 2200 image analyzer. Scale bar = 50 µm. **(B)** Principal component analysis of 12HD vs. 11HC phenotypic data (PCA graph on the right) The analysis shows a separation between the two groups. Each sample is represented by 6 circles each circle is the median of phenotypic data extracted from 20 fields coming from one well, each field contain 10–40 cells, blue circles represent HC samples, red circles represent HD samples. **(C)** (Histogram graphs on the right) represent the distribution of the analyzed PCA data of the two populations in the two dimensions (PC1, PC2), blue histogram represents HC and red histogram represents HD. **(D)** Plot of feature importance in the two PCA dimensions PC1 & PC2, organized from the most important (orange) to the least important feature (black) to the significant difference between the two populations in the PCA analysis.

To determine phenotypic differences by image-based HCA of primary HD fibroblasts, we analyzed 12 HD and matching human control (HC) cell samples. The primary cells from five individuals from each group were plated using a robotic liquid handling unit in 96 well plates in DMEM and 10% FBS and incubated for 24 h at 37°C, 5% CO_2,_ washed, and finally stained with a mix of fluorescent dyes, Hoechst 33,342 (blue), calcein-AM (green), mitotracker (far red), and TMRE (red) in HBSS to label the nuclei, cell cytoplasm, mitochondria, and functional mitochondria, respectively. Automated imaging with a 20x objective of thousands of fluorescently stained cells per sample at the four fluorescence channels was performed under environmental controlled conditions using an IN Cell 2200 Analyzer, and image HCA was performed using this system software as described in the Materials and Methods. Figure 1 shows the results from these experiments. Representative micrographs of the labeled cells for image analysis are shown in Figure 1A. Quantitative analysis of these experiments showed clear segregation of the phenotype between the HD and HC cell samples by principal component analysis (PCA) that gave a 64% variance ratio (Figure 1B). The feature histogram distribution by PC dimension indicates a clear difference between the groups (Figure 1C). Detailed analysis of all significant features from HC and HD fibroblasts samples is shown in Supplementary Figure S1. Together with this we performed a cell by cell analysis to verify the nature of the data distribution (see Supplementary Figure S2) which showed similar results as in Figures 1B, C. As shown in Figure 1D, the most relevant HD phenotypic characteristics were mitochondria features and cell morphology, consistent with previous studies (Costa and Scorrano, 2012). Notably, besides these features, we found that nuclear morphological features are distinctive in the HD cell populations, indicating an HD nuclear phenotype which, to the best of our knowledge, has not been described before.

### Lack of actin cap in skin fibroblast samples from HD patients is correlated with deficient cell motility and low lamin A/C levels

**FIGURE 2:**
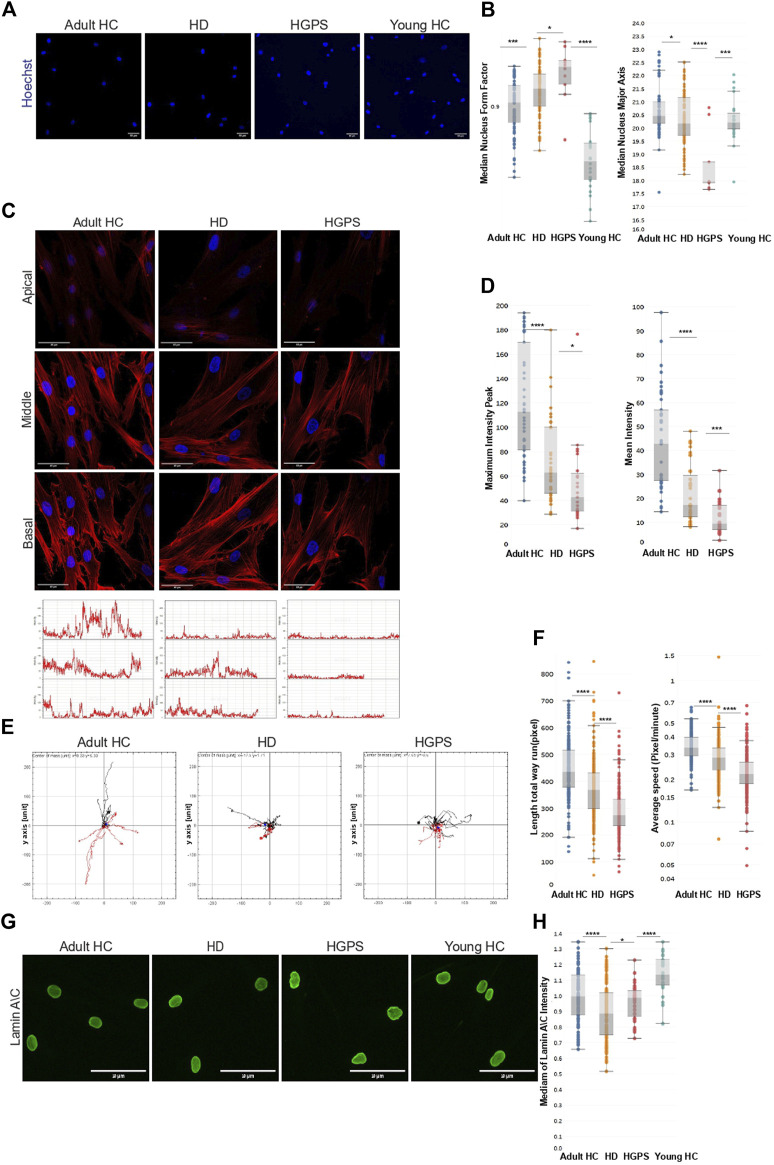
Actin Cap in HD skin fibroblast cells **(A)** Representative images of fixed primary skin fibroblast cells of Adult HC, HD, HGPS, and young HC cells stained with Hoechst to label the cell nuclei and phalloidin to label F-actin, acquired with a 20x magnification using INCell 2200 image analyzer. Scale bar = 50 µm. Cells of indicated groups were segmented automatically using the INCell Developer toolbox software. **(B)** Mann-Whitney’s U test was performed on the medians of the nuclear form factor and nuclear major axis per well (11 HC, 12 HD, 5 HGPS, 4 young HC), ∼500 cells from each skin fibroblast sample using GraphPad 8; the results are presented in box plots using Tableau 2020.3. **(C)** On the upper panel representative confocal immunofluorescence staining images of HD, HC, and HGPS skin fibroblast cells of 3 z-stacks (Basal, Middle, Apical) showing less actin filaments in the apical surface of the HD and the HGPS fibroblast cells. Skin fibroblast cell images were acquired with a 40x magnification using Leica SP8 confocal microscope. Scale bar = 50 μm. Cells were stained with Hoechst to label the cell nuclei and phalloidin to label F-actin of fixed cells. On the lower panel representative intensity graphs of actin in the apical z-stack, each graph represents one nucleus, Y-axis represents intensity levels (0–250), X-axis represents pixels. **(D)** Mann-Whitney’s U test was performed on the medians of the mean intensity of actin fibers, and of the maximum peak of different actin intensity graphs in the apical z-stack of ∼20 cells from each skin fibroblast sample (2 HC, 2 HD, 2 HGPS) showing significant lower measurements in the two parameters in HD and HGPS samples. **(E)** Single-cell random migration analysis of HD and HC skin fibroblast cells shows significant lower motility parameters in HD and HGPS cells compared to HC. Cell tracking analysis was carried out for 21 h at a rate of 1 frame per 10 min. Several parameters of random motility were quantified on (N = 2) of each HD and HC and HGPS skin fibroblast cells. Time-lapse images were acquired in brightfield with a 4x magnification using INCell 2200 image analyzer. Cells of indicated groups were tracked semi-automatically by CellTracker analysis software. After cell tracking, the cells’ paths were plotted and analyzed through The Chemotaxis and Migration Tool (ImageJ plug-in) to create the sun plots. Sun plots show the cell path for representative 15 cells from each population. **(F)** Mann-Whitney’s U test was performed on the medians of different parameters of random motility in HC fibroblast cells compared to the HD and HGPS per cell (2HC, 2HD, 2HGPS), ∼150 cells from each skin fibroblast sample using GraphPad 8, the results are presented in box plots using Tableau 2020.3. **(G)** Representative merged fluorescence images of fixed primary skin fibroblast cells of Adult HC, HD, HGPS, and Young HC cells stained with Hoechst to label the cell nuclei and phalloidin to label F-actin, and Anti-mouse secondary antibody 488 to label Lamin A\C acquired with a 20x magnification using Operetta-high-content screening system, Perkin Elmer. Scale bar = 100 µm. Cells of indicated groups were segmented automatically using the Harmony software for image analysis. **(H)** Mann-Whitney’s U test was performed on the medians of Lamin A\C intensity levels (normalized to HC adult) per well (7 HC- 108 wells, 12 HD- 128 wells, Young HC- 24 wells, 3 HGPS- 36 wells) using GraphPad 8; the results are presented in box plots using Tableau 2020.3.

To investigate this phenotype further, we analyzed all the nuclei images obtained from the above experiment together with newly and similarly acquired nuclear images of primary fibroblasts from five HGPS patients and respective matching controls. The HGPS cells were used as a known reference for disease nuclear morphology (Hale et al., 2008). Results from these experiments (Figures 2A, B) show significant qualitative and quantitative differences in nuclear morphological features (form factor and major axis of the nucleus) between HD and HGPS and their respective adult and young HC groups. Compared to controls, the HD and HGPS cells show significantly higher roundness, which is expressed here as median nucleus form factor. In contrast, the healthy controls show a significant difference in median nucleus major axis, indicating a more oval nuclear morphology than both HD and HGPS cells.

Previous studies showed that nuclear morphological changes are linked to changes in the assembly or absence of a nuclear actin cap (Khatau et al., 2009). To investigate the possibility that the different nuclear morphology in HD cells is linked to an actin cap deficiency, we performed similar experiments as described above but stained the cells after 4% PFA/PBS fixation with Hoechst and phalloidin to label the nuclei and actin filaments respectively. Confocal imaging was applied to measure the phalloidin-labelled actin filaments within the stained nuclear area at three different optical z-section levels – basal, middle, and apical, taken from HD (n = 2), matching HC (n = 2), and HGPS (n = 2) (Figure 2C). Representative images of these cells at the three different z confocal planes, along with linescan analyses of phalloidin fluorescence intensity at the apical level of the cells for three arbitrary nuclei for each group, are shown in Figure 2C upper and lower panels, respectively. Quantitative analysis of the phalloidin mean fluorescence intensity in all apical z-sections analyzed (∼50 nuclei per sample) in each group show significantly higher values in HC compared to HD and HGPS (Figure 2D). These results strongly indicate that actin filaments at the apical side of the nucleus (actin cap fibers) are more readily assembled in HC compared to HD and HGPS cells, although HD cells displayed higher levels than HGPS cells. The significantly low F-actin levels in HGPS cells are in line with their known severe actin cap deficiency (Hale et al., 2008; Khatau et al., 2009).

Based on these findings, we next tested whether the actin cap had an effect on cell motility, as shown in previous studies (Kim et al., 2014). To this end, we performed cell motility analyses on HD (n = 3), HC (n = 3) and HGPS (n = 3) fibroblasts samples using phase contrast time lapse microscopy and image acquisition every 10 min for 21 h (see Supplementary Figure S3 for representative cell migration videos) (tracking of ∼150 cells per sample was performed). As shown by the sunplot cell migration graphs in Figure 2E, both HD and HGPS cells display significantly lower migratory capacity compared to HC cells. Measurements of track distance and average speed of all tracked cells (Figure 2F) confirm the observed migration phenotypes and the differences between HD and HGPS as compared to HC. Together with this we measured the Lamin A\C protein levels by immunofluorescence analysis in the HD and HGPS skin fibroblast samples to investigate apparent phenotype resemblance. Results from these experiments (Figures 2G, H) show significant difference in the intensity levels of Lamin A\C protein between HD and HGPS and their respective adult and young 0HC groups. Compared to their controls, the HD and HGPS cells show significantly lower intensity levels of Lamin A\C. Altogether, these results confirm that aberrant cell motility and Lamin A/C levels of HD cells is correlated with the newly described actin cap deficiency in these cells.

### Classification of different actin cap morphologies in HD skin fibroblast population

**FIGURE 3:**
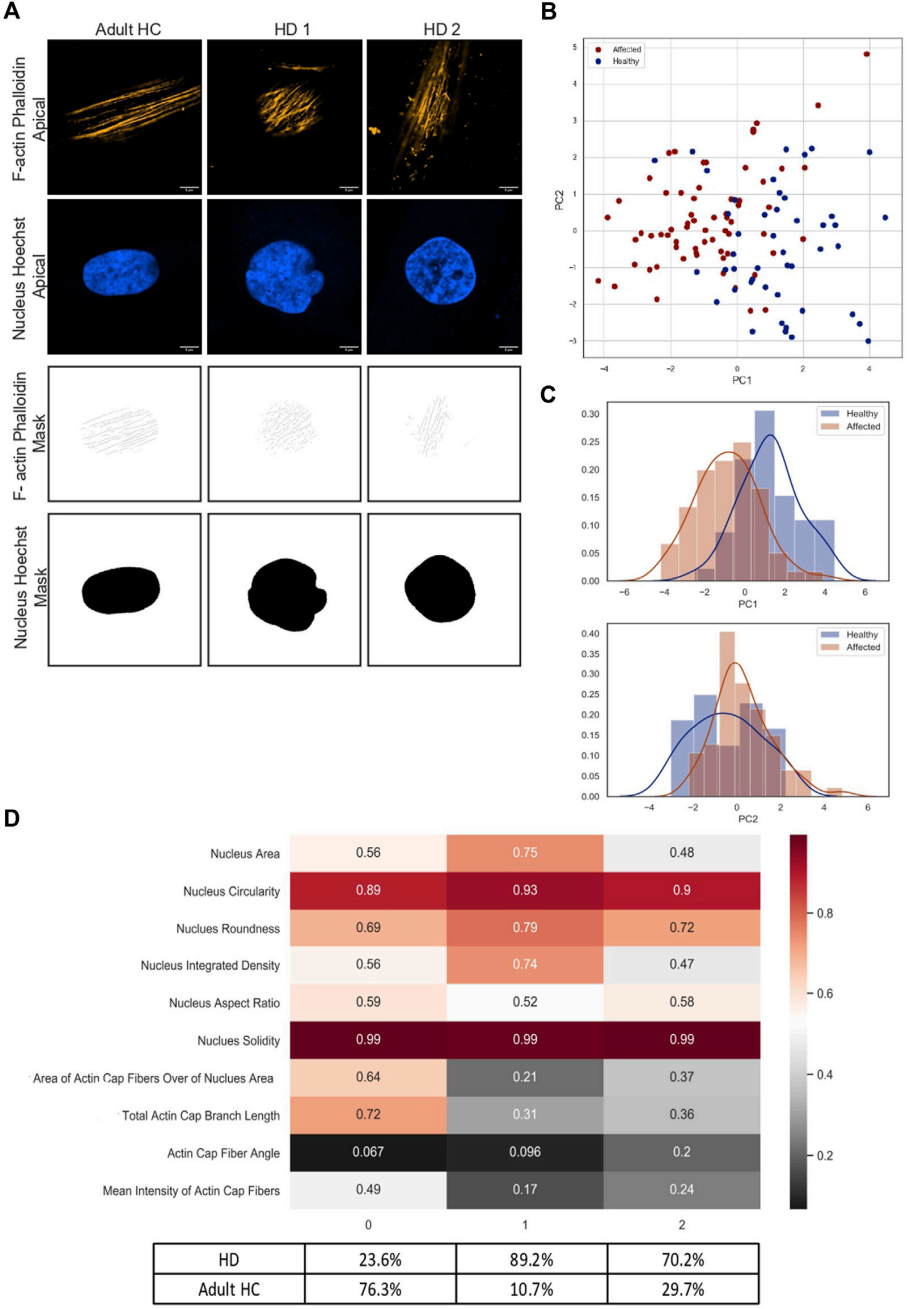
Deeper analysis for the different actin cap morphologies in HD skin fibroblast cells **(A)** Representative confocal immunofluorescence staining images of data acquired under 63x magnification 1.4NA objective and Airyscan module. Cells were stained with Hoechst to label the cell nuclei and phalloidin to label actin filaments of fixed cells. Scale bar = 5 μm For analysis of different morphological clusters of actin cap, for each skin fibroblast cell 3 images were acquired – basal nucleus, apical nucleus, and apical actin. Representative Images of the segmented actin cap fibers and nucleus generated by the WEKA trainable segmentation tool. **(B)** PCA analysis of 8 HD vs. 8 HC actin cap and nucleus morphological features. The analysis shows a significant separation between the two groups. Each sample is represented by 10 morphological features extracted from 1 cell, in total ∼50 cells from each population, blue circles represent HC samples, red circles represent HD samples **(C)** Histogram graphs represent the distribution of the analyzed PCA data of the two populations in the two dimensions (PC1, PC2), blue histogram represents HC and red histogram represents HD **(D)** K-means analysis of three clusters of the same data analyzed by PCA in **(B)**. The values in the plot are the average of the different values of one measurement in specific cluster. Cluster 0 accumulated mainly by HC (Class0: 76.3% HC, 23.6% HD) and Cluster 1, and 2 accumulated mainly by HD cells (Cluster 1: 89.2% HD, 10.7% HC; Cluster 2: 70.2% HD, 29.7% HC).

To further investigate the actin cap phenotype in HD cells and better classify it compared to HGPS cells, which are known for their actin cap deficiency, we developed a machine learning tool for actin cap segmentation using the Trainable Weka segmentation plugin for FIJI (Arganda-Carreras et al., 2017). To this end we performed similar experiments as shown above (see Figure 2C) but images were acquired using higher resolution confocal imaging (63x 1.4NA objective and Airyscan processing) to obtain better description and higher number of the actin cap morphological features (Figure 3A). Phalloidin-labeled actin filaments within the stained nuclear area were analyzed at the apical and basal optical z-section levels from images of 120 cells in total taken from HD (n = 9), matching HC (n = 6), and HGPS (n = 2) samples. Image analysis of HGPS cells produced no segmentation of the actin cap, as expected (see Supplementary Figure S4), and therefore were not included for further analyses. The data obtained from the image segmentation analysis from HC and HD cells in these experiments was analyzed using the script available at https://github.com/disc04/simplydrug. To get a better resolution in actin cap morphology in HD cells, we extracted different morphological features of the actin cap and the nucleus from the images. Detailed analysis of all significant features from HC and HD fibroblasts samples is shown in Supplementary Figure S6. This enabled us to obtain classification within each group, as shown in Figure 3. PCA analysis of these features gave a 55% variance ratio showing a significant difference between the two populations (Figure 3B). The feature histogram distribution by PC dimension indicates a clear difference between the groups (Figure 3C). K-means clustering analysis of three clusters was performed, and results are shown as a heat map (Figure 3D). Cluster 0, which is composed of 23.6% HD cells and 76.4% HC cells, is identified by moderate size and oval nuclei, with the highest actin cap area and highly parallel actin cap fibers with high F-actin intensity levels. Cluster 1, which is composed of 89.3% HD cells and 10.7% of HC cells, is characterized by the largest, more circular nuclei, together with the smallest actin cap area in combination with highly parallel actin cap fibers and low F-actin intensity levels. Cluster 2, which is composed of 70.3% HD cells and 29.7% HC cells, is characterized by the smallest and most circular nuclei, together with a larger actin cap area, compared to those in cluster 1, with non-parallel actin cap fibers and moderate F-actin intensity levels. Overall, these results show that the HD cell population is represented in two different clusters, strongly indicating that the HD actin cap phenotype is heterogeneous, although most of the HD cells analyzed were found in cluster 2. To better understand the different HD actin cap clusters, we analyzed six features describing the actin fiber network at the apical side of the nucleus. These analyses show high correlation between the features (Supplementary Figures S5A, B) which enabled us to reduce the features into one representative feature of the actin cap, namely, actin network complexity. As shown in Supplementary Figure S5C, this feature manages to segregate HD from HC groups, by medium and high values in 11.4% of the HD data and 15.9% of the HC data, respectively. Moreover, low values of actin network complexity are represented in 88.6% of the HD data and 84.1% of the HC data, respectively. These results suggest that the actin network complexity of HD cell population is lower than that of the HC group.

Altogether, these results strongly support the notion that actin cap deficiency in HD is a novel representative cellular phenotype of HD primary fibroblasts, which is characterized differently within the HD sample population as compared to control.

### Image-based high content analysis algorithm for extraction of actin cap like features from 2D images

**FIGURE 4:**
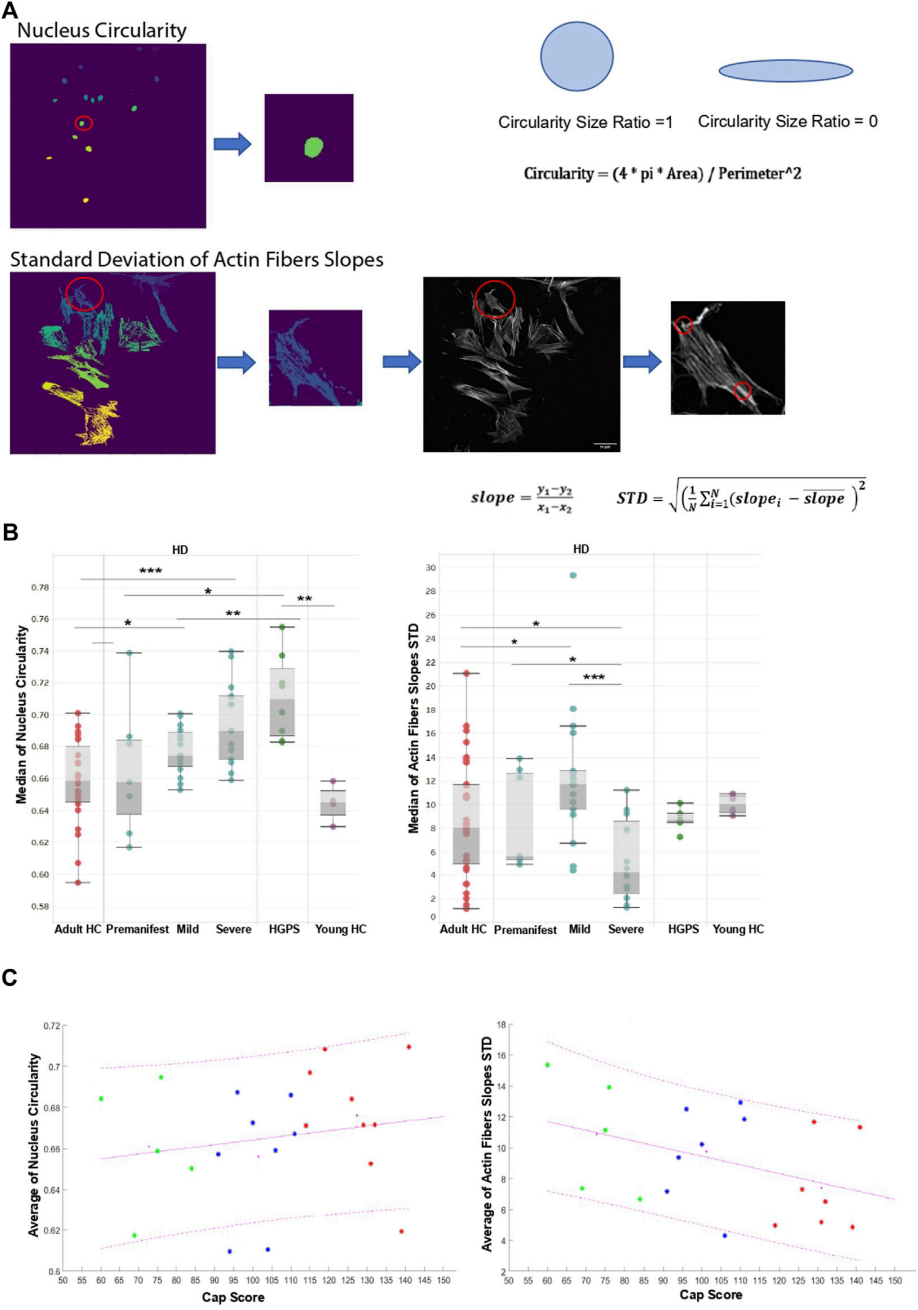
An automatic image based high content analysis algorithm for actin cap like features **(A)** Schematic Representation of the segmentation steps for each feature. For the “Nucleus Circularity” feature the algorithm counts the total number of pixels constructing each nucleus segment over the nucleus image. A binary mask of each nuclear segment is generated and then used to measure the circularity. For the “Standard Deviation of Actin Fibers Slopes” feature, the algorithm segments the cell body (cytoplasm), and a binary mask is applied over the actin fibers image to receive an output image of actin fibers that overlap the specific cell’s cytoplasm. Then, an algorithm for line detection was applied on the output image in order to detect edge points couples of each actin fiber over the cytoplasm. For each actin fiber (“line”) over the cytoplasm, a linear slope was calculated, and a standard deviation value of all slopes was determined to assess the level of actin fibers parallelization over the cytoplasm **(B)** Mann-Whitney’s U test was performed on the medians of different actin cap like features generated from the image analysis tool mentioned above in HC fibroblast cells compared to the HD and HGPS per patient (20 HC, 6 Premanifest HD, 10 Mild HD, 12 Severe HD, 6 HGPS, 4 young HC), ∼500 cells from each skin fibroblast sample using GraphPad 8, the results are presented in box plots using Tableau 2020.3 **(C)** Trend analysis was performed on the average of different actin cap like features in relation to Cap Score of different HD samples divided into three groups (Premanifest in green, Mild in blue, and Severe in red). Magenta lines are represented as least squares lines (solid line - for the average of the data, dashed lines - for the maximum and the minimum of the data) Magenta points are an average point of each group presented in a plot using MATLAB R2021a.

The above results demonstrate a characteristic actin cap deficiency in HD cells. We therefore wished to test whether this phenotype is linked to global actin cell organization and nuclear morphology in the cells, which could be used as a high throughput drug screening assay for HD. To this end we developed a 2D image analysis tool to extract separate features that describe the nuclear morphology and the total actin fiber organization in the cells. Since nuclear elongation and actin cap organization were highly linked (e.g., cluster 0 in Figure 3D), we focused on nuclear circularity and the standard deviation (STD) of actin fibers slopes (see the schematic representation of the image analysis tool in Figure 4A; the image analysis tool is available at https://gitlab.com/maydanw/CellDoctor). This HCA method allowed us to increase the number of fibroblasts samples (a total of 15 HD and 9 HC samples were added to the sample cohort) for analysis as well as the number of cells tested in each sample per experiment. The experiments were performed as described above (see Figure 2). Image analysis using this tool was performed on ∼500 cells per sample of 28 HD, 20 HC, 6 HGPS and 3 young HC fibroblasts (see Table 1; Table 2). The HD samples were analyzed according to their respective CAP score (Abreu et al., 2021) which divides the total number of patients into 3 subgroups: 6 premanifest, 10 mild, 12 severe). Box plot results of the image analysis data representing each of these features is shown in Figure 4B. Using this tool, we found that within the HD cells only mild and severe subgroups show significantly higher values of nuclear circularity compared to matching HC. This is consistent with the levels of HGPS cells that are higher as compared to their matching HC in a similar way as shown above (see Figure 2B). Moreover, HD cells from mild and severe subgroups show higher STD of actin fiber slopes which represent a more disorganized actin fiber arrangement in the cells as compared to matching HC. This is in accordance with what is shown in Figure 3D regarding clustering of the actin cap content and nuclei morphology. Together with this, HGPS cells show higher organization of the actin fibers in the cells, confirming the above detailed confocal data analysis (see cluster one in Figure 3D), which shows that the fewer the number of actin cap fibers, the more organized they are. Therefore, in a similar way here in Figure 4B, the standard deviation of the actin fiber slopes is lower in the matching HC compared to mild and severe HD subgroups. Interestingly, within the HD group data there is a significant difference between premanifest, mild and severe subgroups while the premanifest subgroup of HD cells show no significant difference if compared to HC cells. Figure 4C show the trend analysis of the two nuclear circularity and actin fibers slope STD features in relationship with the cap score of each of the 28 HD samples (premanifest subgroup in green; mild subgroup in blue; severe subgroup in red). The least square lines in both plots indicate the data tendency in relationship with the HD sample cap score. While the nuclear circularity feature increases with the HD cap score the actin fibers slope STD feature decreases. Altogether, these results validate the use of our image HCA tool for actin content and organization in primary skin fibroblasts of HD patients to identify actin cap related phenotype in these cells according to the patients’ disease severity score.

To strengthen the morphological differences coming from the extracted features of the 2D image analysis algorithm with a biological evidence a Lamin A\C immunofluorescence analysis was done on the different HD subgroups (4 Premanifest, 4 Mild, 4 Severe) compared to 7 healthy controls and to 3 HGPS samples see Supplementary Figure S7. Showing significant difference between the HD subgroups.

**FIGURE 5:**
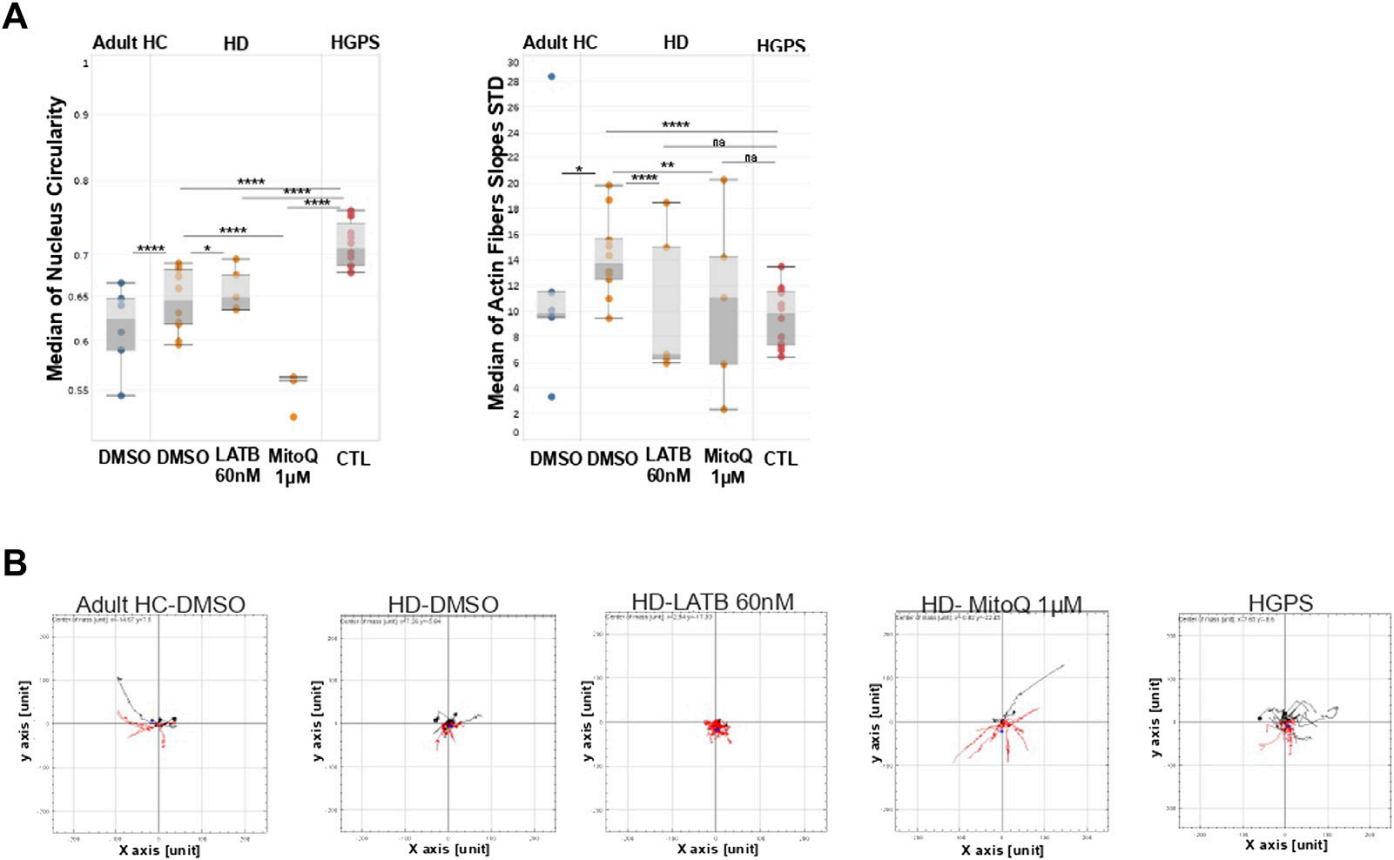
Effect of MitoQ and Latrunculin B on the actin cap in HD Fibroblast compared to HC and HGPS **(A)** Mann-Whitney’s U test was performed on the medians of different actin cap like features generated from the image analysis tool mentioned above in HC fibroblast cells compared to the HD and HGPS per field (3 HC, 5 HD, 2 HGPS), ∼200 cells from each skin fibroblast sample using GraphPad 8, the results are presented in box plots using Tableau 2020.3 **(B)** Cell tracking analysis was carried out in the same conditions mentioned in materials and methods section. Sun plots show the cell path for representative ∼10 cells from each population. Graphs were generated by The Chemotaxis and Migration Tool (ImageJ plug-in).

These results prompted us to test the robustness of the distinctive actin cap HD phenotype as a new predictive tool by treating the cells with two different pharmacological agents, latrunculin B that blocks actin cap formation at low concentrations (Kim et al., 2014), and Mitoquinone mesylate (MitoQ), an antioxidant agent targeted to mitochondria to protect from oxidative stress. For functional treatment effects we performed in parallel cell motility time lapse microscopy assays as described (see Figure 2E). We hypothesized that latrunculin B treatment of HD cells would lead to a phenotype similar to that of HGPS cells that lack an actin cap completely. In contrast, we hypothesized that MitoQ treatment might recover the actin cap phenotype in HD cells due to the potential improvement in the known mitochondrial distress in HD cells (Carmo et al., 2018). To this end we performed experiments similar to those described above, where HD cells from five patients were treated for 24 h with either 60 nM latrunculin B or with 1 µM MitoQ, or DMSO (1:1000 v/v) as control. Matching HC (n = 3) and HGPS (n = 2) fibroblasts were used as controls. The effects of latrunculin B and MitoQ treatments on actin and nuclei morphology features in HD fibroblast cells are shown in Figure 5A. Interestingly, these two different drugs exert an opposite effect on the cells, where Latrunculin B treatment resembles HGPS nuclear circularity and shows a significant difference from HD DMSO control cells. In contrast, MitoQ led to a significant effect on nuclei circularity in HD treated cells compared to DMSO and similar to those of HC cells. In addition, Latrunculin B decreased the actin fiber slope standard deviation of HD cells compared to the DMSO control, indicating more parallel actin fiber organization despite the decrease in intensity. This effect resembles the phenotype that defines cluster one in Figure 3D. MitoQ in these experiments also affected the actin fiber slope standard deviation of HD cells significantly, but in contrast the phenotype resembles cluster 0 in Figure 3D which is representative of the HC.

Altogether, from these results we conclude that Latrunculin B at low concentrations exacerbates the actin cap phenotype of the HD cells, making it more similar to the actin cap phenotype of HGPS cells. In contrast, MitoQ improves the actin cap phenotype significantly in HD cells, making it more similar to the actin cap phenotype of HC cells. Remarkably, MitoQ also restored the motility of HD cells in all the measured parameters whereas Latrunculin B did not (see Figure 5B). Moreover, the opposite effects of the two drugs on cell motility demonstrate the direct functional link between their respective effects on actin, actin cap, and nuclear morphologies and cell motility. Altogether, these results strongly support the use of the actin cap associated features and cell motility assay to screen and evaluate drugs on HD patient cells for potential personalized treatment of HD patients in the clinic.

## Discussion

Although the HD mutation in the Htt gene is a well-characterized cause of disease, the mechanism of disease in the different tissues and organs remains to be elucidated. Brain pathology has become a hallmark of HD, but critical mass of new studies suggest that HD is a multi-system challenging disorder, contributing to the patient’s individual disease progression and disease severity independently of the high number of CAG repeats in the mtHtt (Sathasivam et al., 1999; Mielcarek, 2015). Therefore, it is important to characterize the phenotypic implications of mtHtt expression in cells other than neurons, such as primary HD fibroblasts, and try to connect them with specific cellular biological functions affected in the HD patients’ cells. To this end we applied an unbiased high throughput cell-based image analysis approach to characterize an HD phenotype in these cells. Our results show that primary HD patients’ fibroblasts exhibit a distinctive cellular phenotype that allows accurate classification from HC by image-based HCA tools. This analysis on 16 HD fibroblasts samples compared to 18 matching HC, strongly supports the robustness of such phenotypic classification. The HD cell phenotype is characterized by differences in cell morphology and mitochondrial features previously described in other HD models (Squitieri et al., 2010; Carmo et al., 2018). In addition, we found, for the first time, that significant nuclear morphological features are distinctive in the HD cell population (see Figure 1). A deeper analysis into the nuclear morphology of the cell by confocal microscopy allowed us to test the known link between nuclear roundness and the actin cap morphology (Figure 2) (Khatau et al., 2009). These experiments were performed using both HC fibroblasts as positive control and HGPS fibroblasts as reference for actin cap depletion control, since these patient cells are known to have a severe actin cap and concomitant nuclear morphology abnormalities (Booth-Gauthier et al., 2013). The confocal morphometric results strongly indicate that HD cells resemble more the HGPS nuclear morphology together with an associated actin cap deficient phenotype than the HC, suggesting that HD shares aspects associated with HGPS laminopathy. Further support for the HD phenotype comes from our mechanistic actin cap-related experiments, measuring cell migration for 24 h by time-lapse microscopy (Figure 2) (Kim et al., 2014). The migratory capacity of the HD cells confirms that their actin cap deficiency is correlated with their cell motility, which falls also in between the HC and HGPS control groups.

Studies show that mutant Htt interferes with actin dependent cellular remodeling. Mutant Htt fragments interact abnormally with actin binding proteins in HEK293 cells transfected with Htt Exon 1 (Munsie et al., 2011), and in mouse striatal neuron-derived cell line (STHdh) expressing full-length endogenous levels of either wild-type (STHdhQ7/Q7), or mutant (STHdhQ111/Q111) (Angeli et al., 2010). Different studies support the idea that Htt is involved in regulating adhesion and actin dependent functions including those involving α-actinin (Tousley et al., 2019) – an actin-binding protein and one of the components of the actin cap (Djinovic´ et al., 1999; Sjöblom et al., 2008). α-Actinin is recruited to focal complexes, where it provides a structural link between integrins and actin microfilaments (Sjöblom et al., 2008; Meacci et al., 2016). An interactome analysis of huntingtin in mouse brain models showed that Htt is associated with α-actinin-1, -2 and -4 both in wild type and Htt mutant huntingtin (Culver et al., 2012; Shirasaki et al., 2012). Follow up studies validated these results through immunoprecipitations using exogenously expressed proteins, immunofluorescence co-localization, and proximity ligation assay, showing a functional interaction of Huntingtin with isoforms of α-actinin in human primary fibroblasts and neurons (Tousley et al., 2019). This study, along with work of others (Greenwood et al., 2000; Guo et al., 2006; Kovac et al., 2013), proposed a model in which Huntingtin regulates α-actinin-1 localization and couples growth factor signaling with actin polymerization with bundling functions at new sites of adhesion. This evidence regarding htt and α-actinin subtypes strongly supports the possibility in HD patients’ fibroblasts that actin cap might be aberrated due to abnormal binding between α-actinins and htt due to mt-htt expression.

Lamin B protein is one of the two subgroups that compose the lamin family which not only provide structural support to the nuclear envelope membrane, but is also involved in a wide variety of cell functions and processes (Hozak et al., 1995). Lamin B is expressed in almost all cell types independently of their differentiation state (Verstraeten et al., 2007), suggesting its critical role in mammalian cell survival (Harborth et al., 2001). It is also known that lamin B binds directly to F-actin that forms the actin cap (Simon et al., 2010). Thus, altered levels of lamin B may have a direct effect on the formation, stabilization, and organization of the actin cap. Very recent work demonstrated a new pathogenic mechanism for HD by showing an increase in the Lamin B1 protein levels in HD brains in a neuron-type specific manner, which is correlated with alterations in nuclear morphology and nucleocytoplasmic transport (Alcalá-Vida et al., 2021).

We also show here that the actin cap phenotype in HD fibroblast cells is correlated with a distinctive nuclear morphology which allowed us to develop an image high throughput analysis tool of the actin content and organization in the nucleus area and nuclear morphology phenotype using 20X low power magnification. This tool allowed us to increase the amount of data for analysis which in turn gave us grading power to characterize and identify the morphological cell-based data with the respective HD sample’s cap scoring. Moreover, these results support that actin cap morphological features are reliable HD cell biomarkers. Interestingly an important conclusion from the actin cap/cap scoring analyses of the data is that the actin cap phenotype in HD cells is acquired during the symptomatic phase of the disease. Following by the evidence that there is no actin cap alteration within the HD premanifest subgroup in relation with the other two HD symptomatic subgroups (mild and severe) which are significantly different from HC. Furthermore, the HD severe subgroup showed no significant difference from the HGPS group which is hallmark for actin cap defect.

The relative simplicity of this method should allow using this HD cell phenotype biomarker as reliable indicator to test or screen drug effects for patients with HD. This observation could allow us to classify/treat the patients in the future according to their cell phenotype severity level. The use of known pharmacological agents such as Latrunculin B and MitoQ to test their expected opposite effects on the HD actin content and organization in the nucleus area and nuclear morphology allowed us to test the drug screening potential of the assay. From these experiments, it becomes clear that while Latrunculin B exacerbates the HD cell phenotype by blocking actin polymerization, MitoQ, which reduces oxidative stress, improves significantly all the morphological features as well as cell migration parameters in the HD cells. Interestingly, these results strongly indicate that the mitochondrial function may regulate the actin cap/nuclear system and concomitant cell motility. Aberrant mitochondrial activity, possibly by increased reactive oxygen species (ROS) production, may be responsible for the actin cap phenotype in HD cells which can be reversed by MitoQ treatment that also recovers functional motility in HD patients’ cells.

Our results strongly support the power of the image based phenotypic HCA assay in discovering new personalized biomarkers in HD. In addition, they also support the use of primary skin fibroblasts from HD patients as personalized model for disease phenotype classification based on linked actin cap/nuclear morphologies that functionally affect cell migration, which can be recovered by pharmacological agents. Altogether, our results and the HCA phenotypic tool used to describe the HD cell phenotype, together with application of machine learning approaches both for improving disease assessment, as well as exploration of novel parameters open the door for future drug testing or drug screening campaigns directly using the HD patient derived cells.

## Data Availability

The raw data supporting the conclusions of this article will be made available by the authors, without undue reservation.
