# Supplementary figures and images for: Perturbed actin cap as a new personalized biomarker in primary fibroblasts of Huntington’s disease patients
Source: Front Cell Dev Biol. 2023 Jan 18;11:1013721. doi: 10.3389/fcell.2023.1013721 (PMC9889876; doi:10.3389/fcell.2023.1013721)

## Slide 1
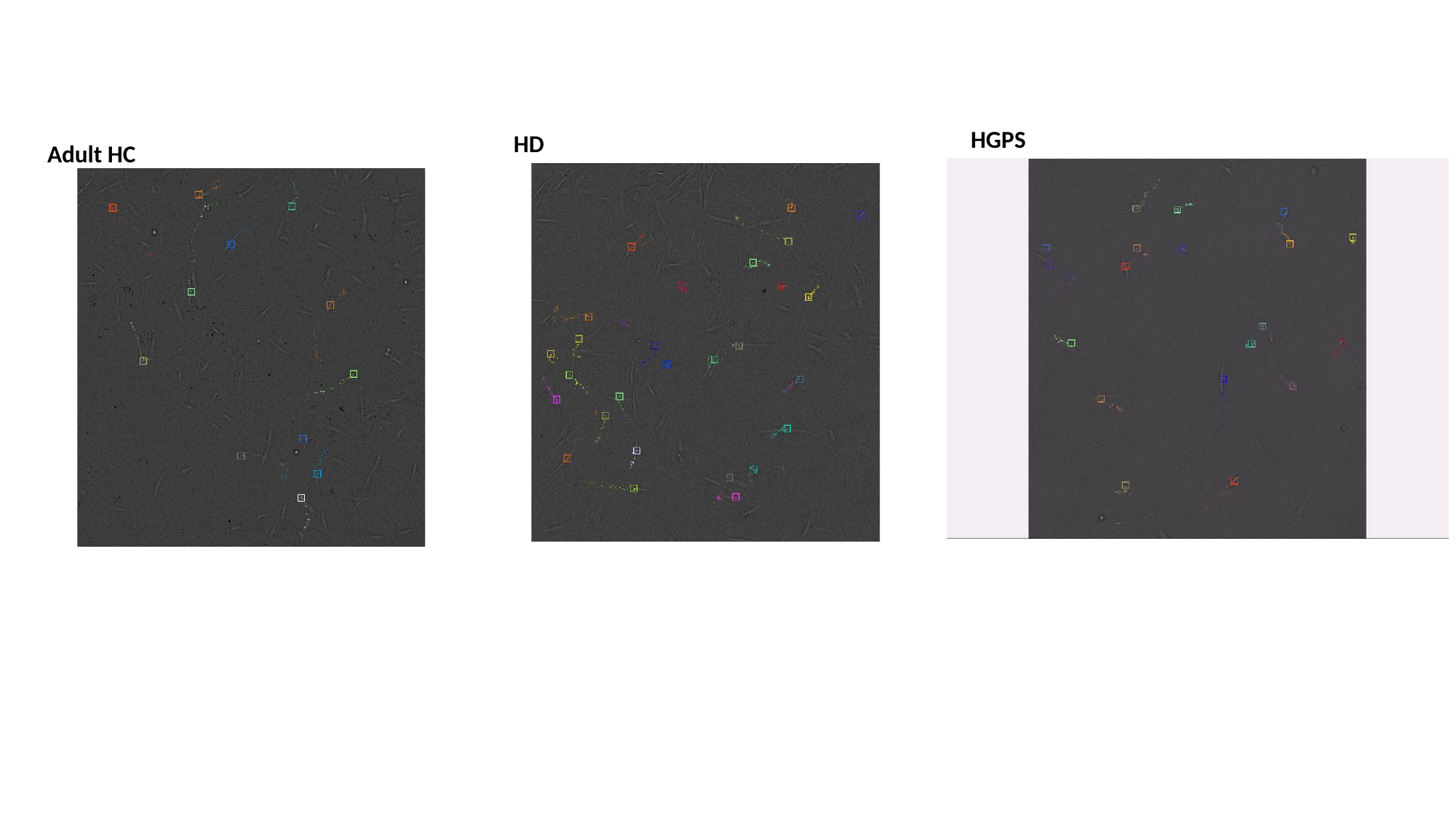

HGPS
HD
Adult HC

Supplement: Supplementary file 1 [file Presentation1.PPTX]
