# Supplementary material for: Perturbed actin cap as a new personalized biomarker in primary fibroblasts of Huntington’s disease patients
Source: Front Cell Dev Biol. 2023 Jan 18;11:1013721. doi: 10.3389/fcell.2023.1013721 (PMC9889876; doi:10.3389/fcell.2023.1013721)
Supplement: Supplementary file 2 [file DataSheet1.PDF]

S.1

A

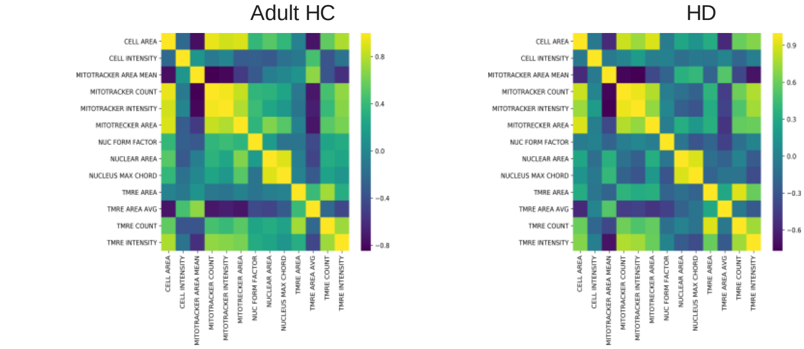

Adult HC
HD
CELL AREA
CELL INTENSITY
MITOTRACKER AREA MEAN
MITOTRACKER COUNT
MITOTRACKER INTENSITY
MITOTRECKER AREA
NUC FORM FACTOR
NUCLEAR AREA
NUCLEUS MAX CHORD
TMRE AREA
TMRE AREA AVG
TMRE COUNT
TMRE INTENSITY

B

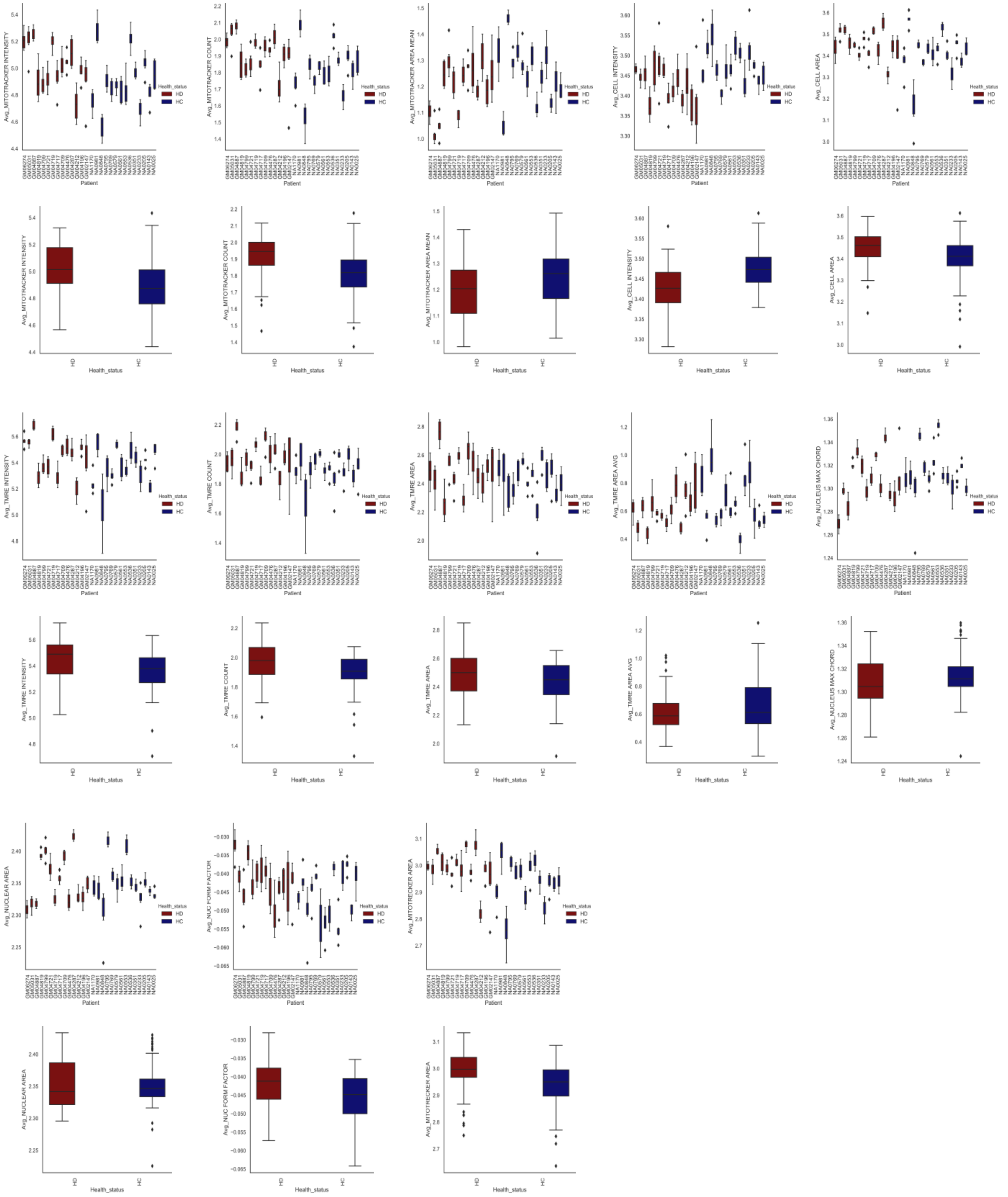

Avg_MITOTRACKER INTENSITY
Avg_MITOTRACKER COUNT
Avg_MITOTRACKER AREA MEAN
Avg_CELL INTENSITY
Avg_CELL AREA
Avg_TMRE INTENSITY
Avg_TMRE COUNT
Avg_TMRE AREA
Avg_TMRE AREA AVG
Avg_NUCLEUS MAX CHORD
Avg_NUCLEAR AREA
Avg_NUC FORM FACTOR
Avg_MITOTRECKER AREA
Patient
Health_status
HD
HC

C

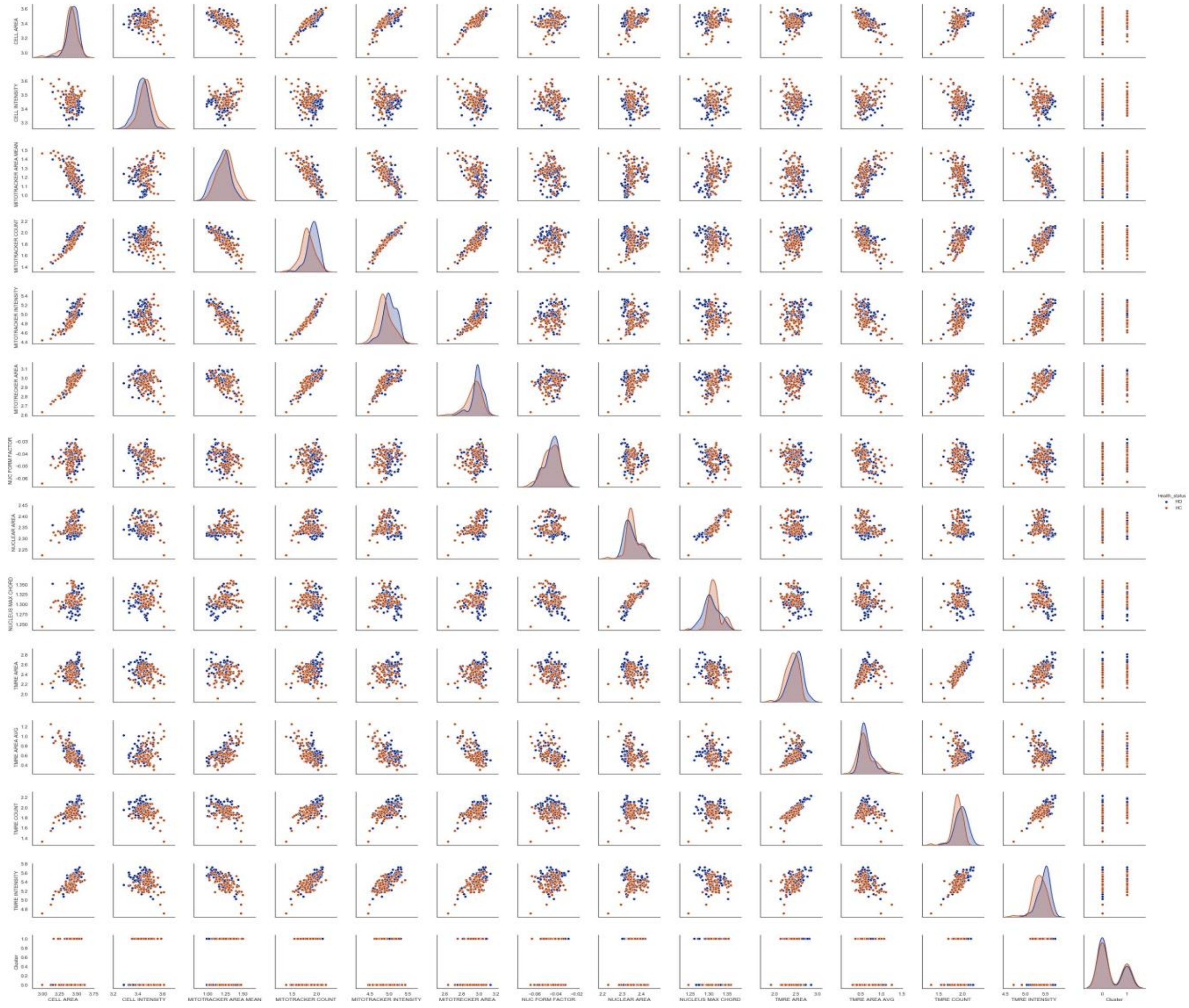

<u>S1: Detailed high content image-based analysis of primary skin fibroblast samples of HD patients compared to HC</u>

(A.1) Correlation heat map of morphological features of 12 HD vs. 11 HC primary skin fibroblast samples; the correlation coefficients are color-coded from deep blue (−1) to yellow (1). (B) Box plots of the average of phenotypic data per patient and per group. The data was extracted from 6 wells for each sample, each well containing 20 fields, each field containing 10-40 cells, blue represents the HC samples and red represents the HD samples. (C) Seaborn pairplot of the morphological data. The point colors correspond to the groups HD in blue and HC in orange. The plot is based on samples of 84 points coming from each group.

S.2

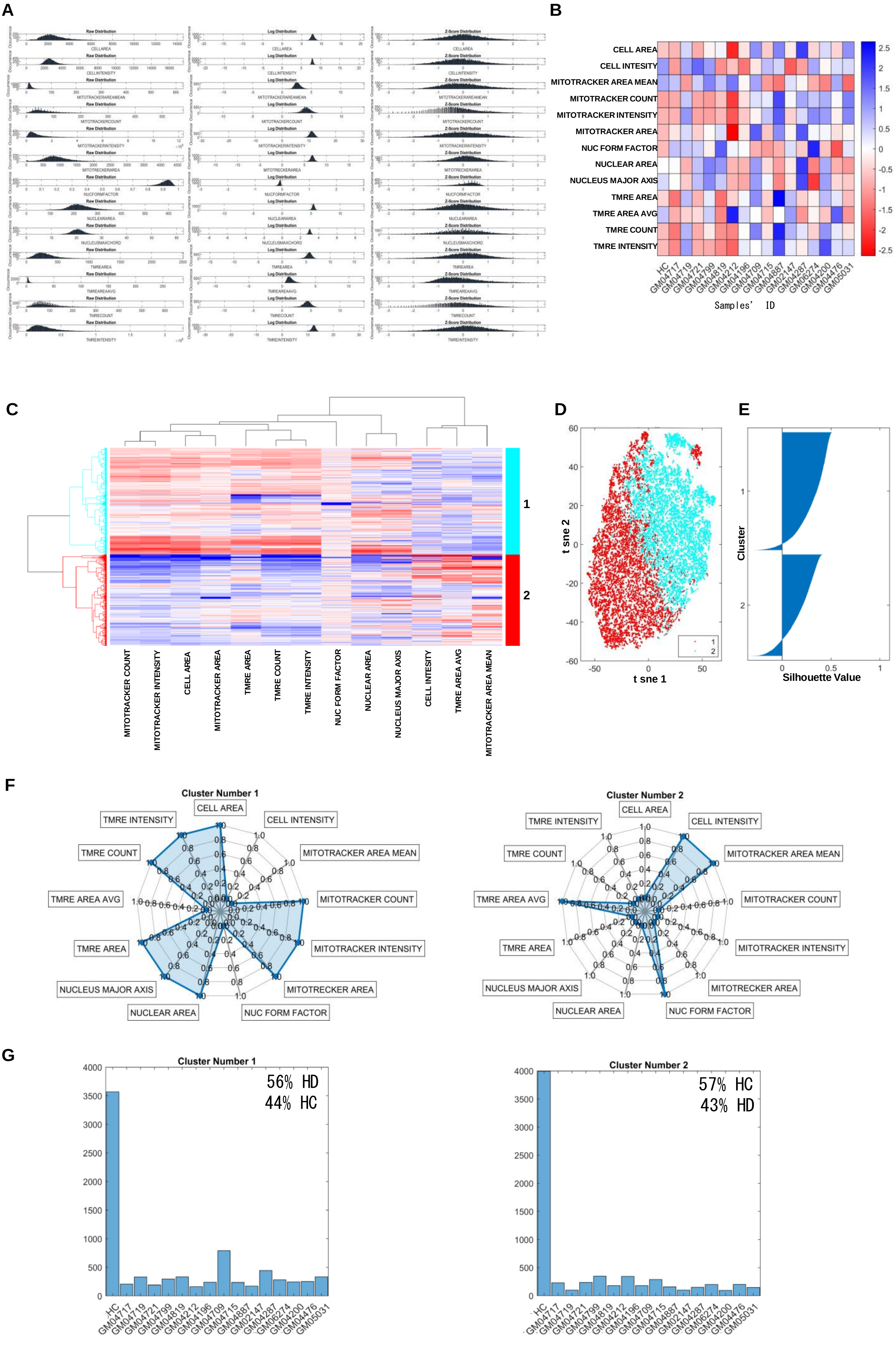

A
Raw Distribution
Log Distribution
Z-Score Distribution
B
CELL AREA
CELL INTESITY
MITOTRACKER AREA MEAN
MITOTRACKER COUNT
MITOTRACKER INTENSITY
MITOTRACKER AREA
NUC FORM FACTOR
NUCLEAR AREA
NUCLEUS MAJOR AXIS
TMRE AREA
TMRE AREA AVG
TMRE COUNT
TMRE INTENSITY
HC GM04717 GM04719 GM04721 GM04799 GM04819 GM04212 GM04196 GM04709 GM04715 GM04887 GM02147 GM04287 GM06274 GM04200 GM04476 GM05031
Samples' ID
C
MITOTRACKER COUNT
MITOTRACKER INTENSITY
CELL AREA
MITOTRACKER AREA
TMRE AREA
TMRE COUNT
TMRE INTENSITY
NUC FORM FACTOR
NUCLEAR AREA
NUCLEUS MAJOR AXIS
CELL INTESITY
TMRE AREA AVG
MITOTRACKER AREA MEAN
D
t sne 1
t sne 2
E
Cluster
Silhouette Value
F
Cluster Number 1
Cluster Number 2
CELL AREA
CELL INTENSITY
MITOTRACKER AREA MEAN
MITOTRACKER COUNT
MITOTRACKER INTENSITY
MITOTRECKER AREA
NUC FORM FACTOR
NUCLEAR AREA
NUCLEUS MAJOR AXIS
TMRE AREA
TMRE AREA AVG
TMRE COUNT
TMRE INTENSITY
G
Cluster Number 1
56% HD
44% HC
Cluster Number 2
57% HC
43% HD

Note for S2: To investigate deeper into the image based -HCA data, cell-by-cell analysis was performed as follows: After the raw data was normalized by log scaling and z-scoring (S2A) an hierarchical cluster analysis was applied which is an unsupervised classification of patterns (cell-by cell observations and feature vectors) into groups. This method groups similar objects into clusters based on measuring the distance between the clusters based on the 'cityblock' distance method along with the 'ward' linkage method which predicted only two clusters (S2 C) that according to the silhouette scoring analysis the two clusters are distinct one from another significantly (S2 E). To identify the two clusters a radar plot analysis showing the values of the features in each cluster and a bar plot showing the number of cells coming from each skin fibroblast sample was done on each cluster (S2 F&G). These analyses showed a similar behavior of the data to the results from the analysis per well. The cell-by-cell data divided by two clusters (Cluster 1 – the HD cluster with majority of HD cells, and Cluster 2 – the HC cluster with majority of HC cells) where each cluster shows the same morphological profile as the data showed in Figure 1 and S1.

S2: Deeper cell-by-cell high content image based analysis of primary skin fibroblast samples of HD patients compared to HC

For cell-by-cell analysis all the features normalized through a normalization process as follows (A) Raw data -> log scaling that computes the log of the raw values and compressed is into a narrow range helping to improve a linear model performance-> Z-Score normalization calculated by: $x' = \frac{x-\mu}{\sigma}$; μ is the mean and σ is the standard deviation. (B) Heatmap of the normalized features correlated to each skin fibroblast sample, each square represents the averaged value of the cells in a specific feature for each sample, the HC column represent the average value for the cells of all healthy controls. (C) Clustergram of a hierarchical clustering analysis calculated by 'cityblock' distances and 'ward' linkage which predicted two clusters (Cluster 1 in blue, Cluster 2 in red. (D) T-SNE plot displaying the cell-by-cell data divided into the clusters (Cluster 1 in red, Cluster 2 in blue). (E) Silhouette plot showing the silhouette values for each cell in the two clusters that indicates how similar an object is to its own cluster (cohesion) compared to other clusters (separation). The silhouette ranges from −1 to +1, where a high value indicates that the object is well matched to its own cluster and poorly matched to neighboring clusters. (F) Radar plots representing the values of each feature in each cluster. (G) Bar plots showing the number of cells from each skin fibroblast sample in each cluster; Cluster 1 is: 56% HD cells and 44% HC cells, Cluster 2 is: 57% HC cells and 43% HD cells.

S3: Cell migration videos

(A) Representative cell migration videos with marked migration path for the analyzed cells (each cell marked with different color) of the three groups Adult HC, HD and HGPS.

S.4

A

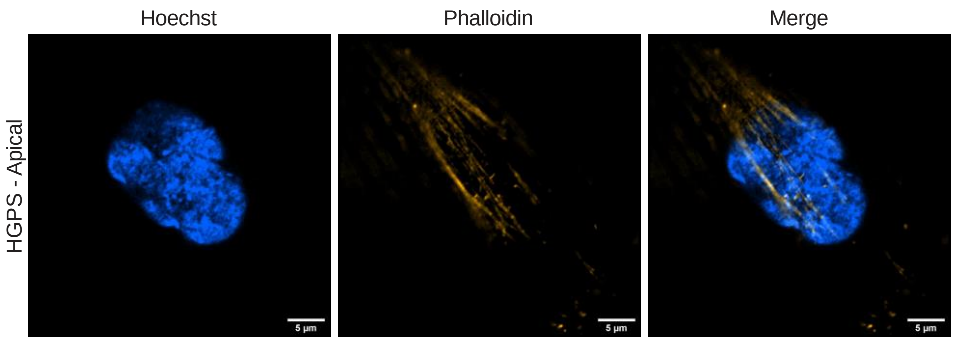

<u>S4: Deeper analysis for the different actin cap morphologies in HD skin fibroblast cells</u> Representative confocal immunofluorescence staining images of data acquired under 63X magnification 1.4NA objective and Airyscan module for HGPS primary fibroblasts in the apical plane. Cells were stained with Hoechst to label the cell nuclei and phalloidin to label actin filaments of fixed cells. Scale bar = 5µm. Actin cap fibers were not detected by WEKA segmentation tool in HGPS fibroblast.

S.5

A

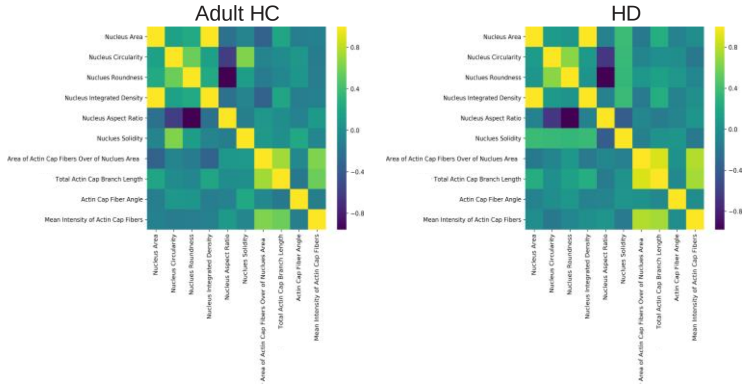
Adult HC
HD
Nucleus Area
Nucleus Circularity
Nucleus Roundness
Nucleus Integrated Density
Nucleus Aspect Ratio
Nucleus Solidity
Area of Actin Cap Fibers Over of Nucleus Area
Total Actin Cap Branch Length
Actin Cap Fiber Angle
Mean Intensity of Actin Cap Fibers
0.8
0.4
0.0
−0.4
−0.8

B

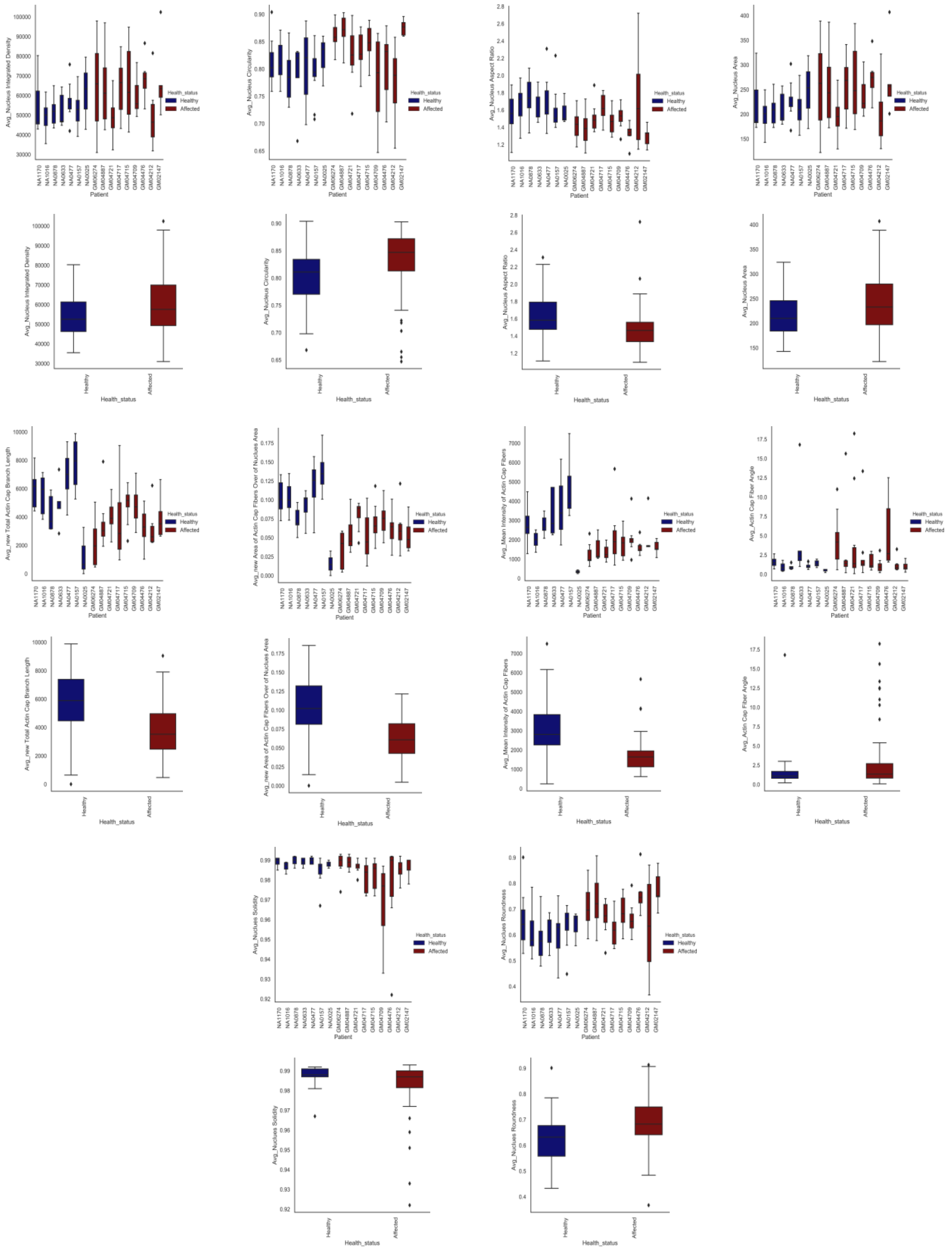
Avg_Nucleus Integrated Density
Avg_Nucleus Circularity
Avg_Nucleus Aspect Ratio
Avg_Nucleus Area
Avg_new Total Actin Cap Branch Length
Avg_new Area of Actin Cap Fibers Over of Nucleus Area
Avg_Mean Intensity of Actin Cap Fibers
Avg_Actin Cap Fiber Angle
Avg_Nucleus Solidity
Avg_Nucleus Roundness
Health_status
Healthy
Affected
Patient
NA1170
NA1016
NA0878
NA0633
NA0477
NA0157
NA0025
GM06274
GM04887
GM04721
GM04717
GM04715
GM04709
GM04476
GM04212
GM02147

C

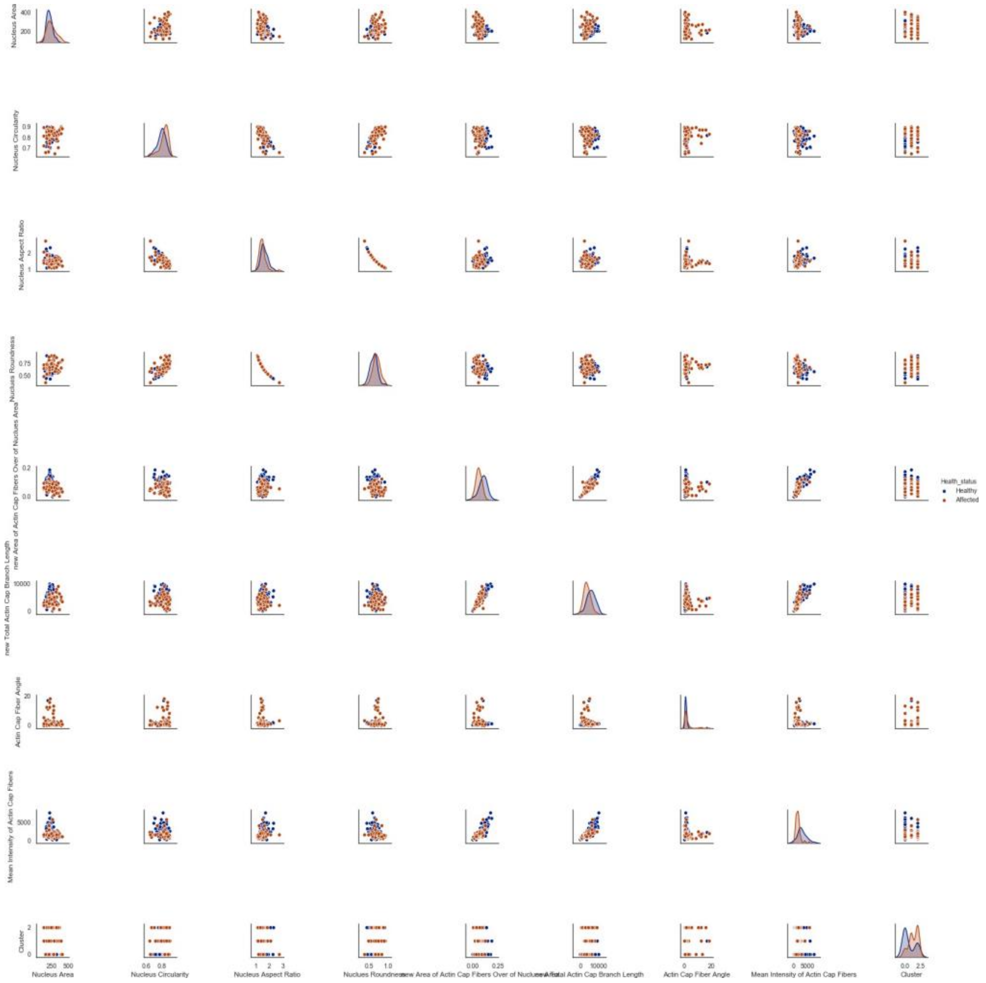

Nucleus Area
Nucleus Circularity
Nucleus Aspect Ratio
Nucleus Roundness
new Area of Actin Cap Fibers Over of Nucleus Area
new Total Actin Cap Branch Length
Actin Cap Fiber Angle
Mean Intensity of Actin Cap Fibers
Cluster
Health_status
Healthy
Affected
Nuclues Roundness

D

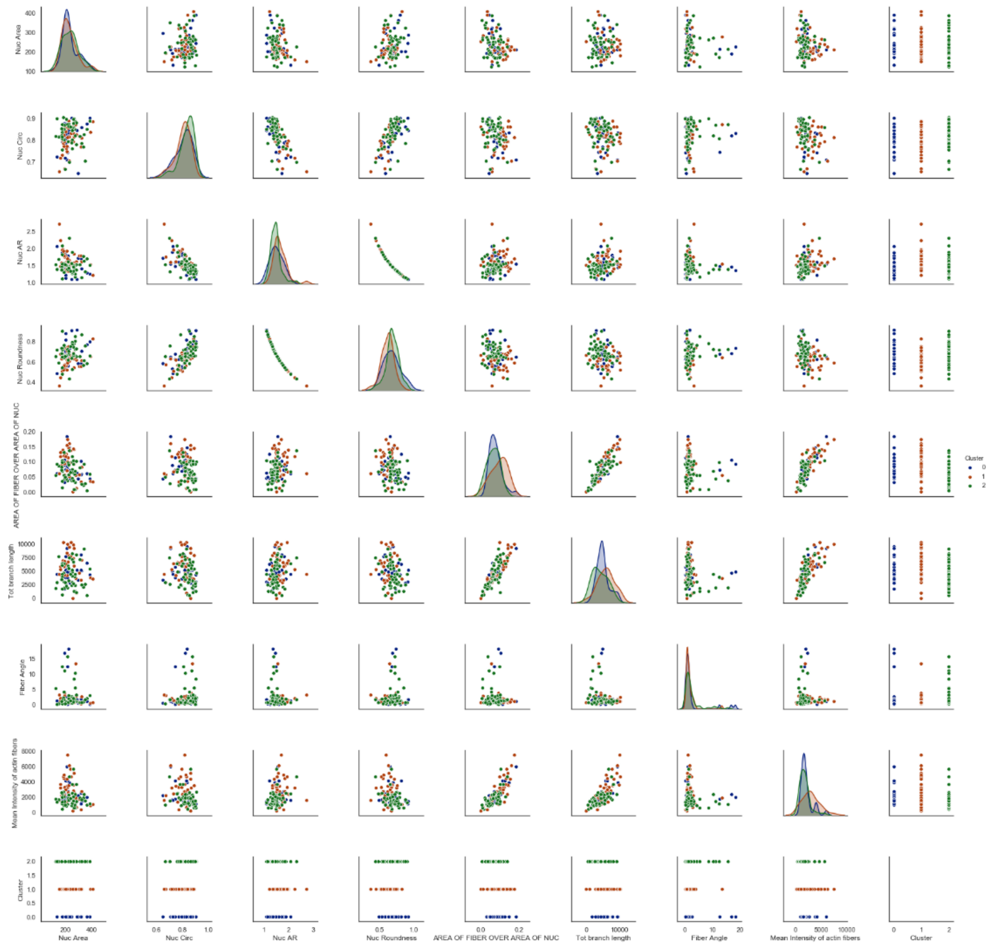

Nuc Area
Nuc Circ
Nuc AR
Nuc Roundness
AREA OF FIBER OVER AREA OF NUC
Tot branch length
Fiber Angle
Mean Intensity of actin fibers
Cluster
Cluster
0
1
2

S5: Detailed analysis for actin cap morphology in HD skin fibroblast cells

(A) Correlation heat map of actin cap filaments morphological features of 9 HD vs. 7 HC primary skin fibroblast samples; the correlation coefficient is color coded from deep blue (−1) to yellow (1). (B) Seaborn pairplot of the morphological data. The point colors correspond to the groups HC in blue and HD in orange. The plot is based on samples of ~11000 points coming from each group. (C) Bar chart representing the percentage of each population HC in blue and HD in red in the branching feature divided into three classes (low, medium, high).

S.6

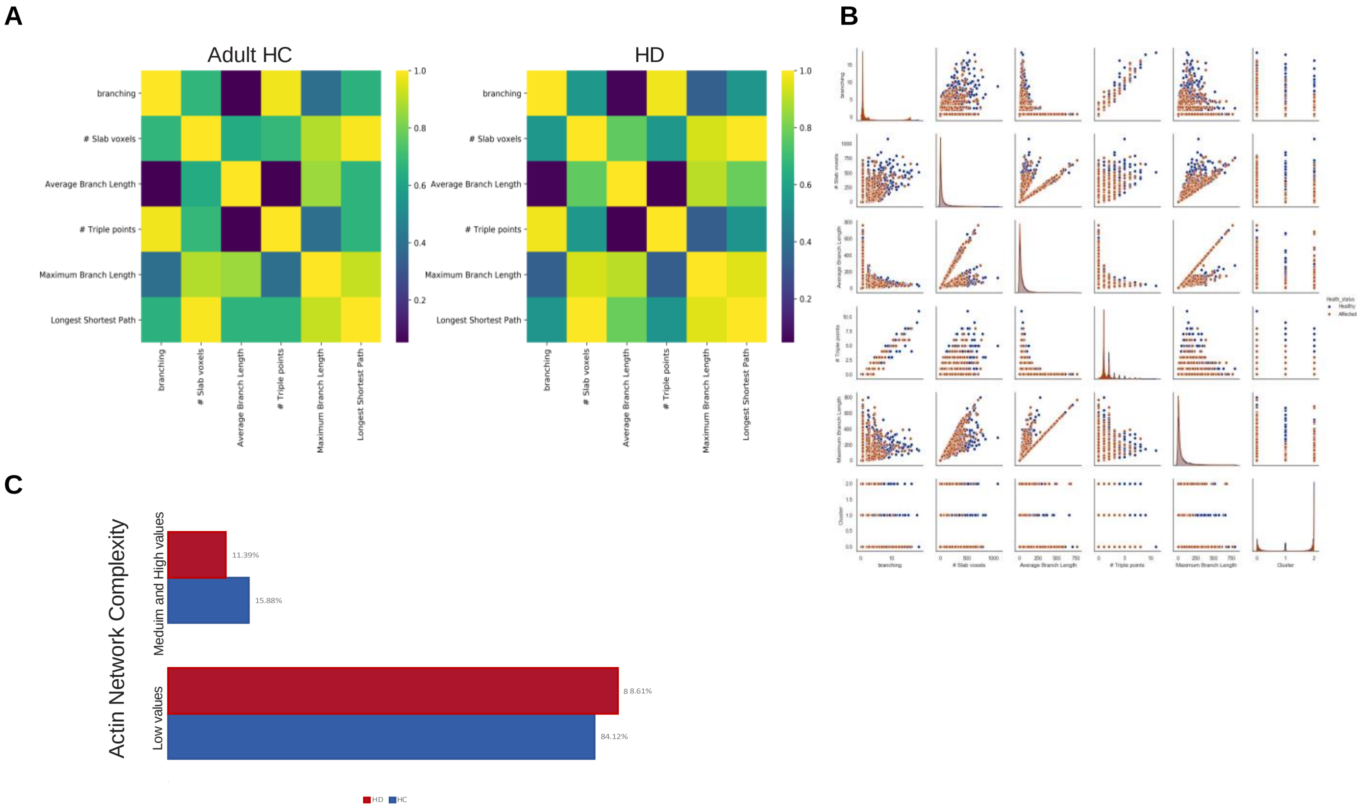

<u>S6: Detailed analysis for actin cap morphological cluster in HD skin fibroblast cells</u>

(A) Correlation heat map of actin cap and nucleus morphological features of 9 HD vs. 7 HC primary skin fibroblast samples; the correlation coefficient is color coded from deep blue (−1) to yellow (1). (B) Box plots of the average of phenotypic data per patient and per group. The data was extracted from ~50 cells from each group, blue represents the HC samples and red represents the HD samples. (C) Seaborn pairplot of the morphological data. The point colors correspond to the groups HC in blue and HD in orange. The plot is based on samples of ~50 points coming from each group. (D) Seaborn pairplot of the morphological data in 3 clusters. The point colors correspond to the clusters, cluster 0 in blue, cluster 1 in orange, and cluster 2 in green. The plot is based on samples of ~50 points coming from each group.

S.7

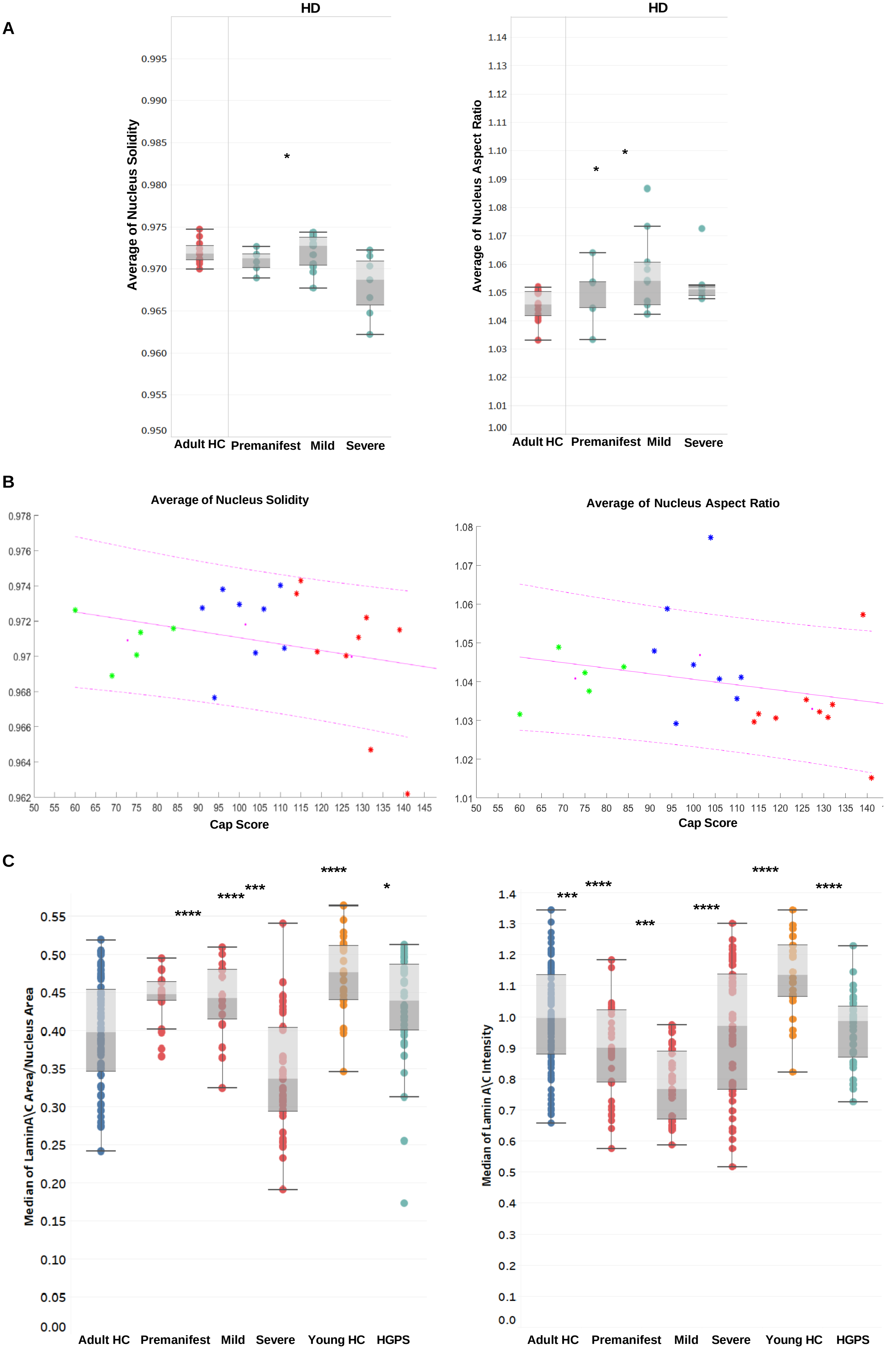
A
HD
Average of Nucleus Solidity
Adult HC
Premanifest
Mild
Severe
HD
Average of Nucleus Aspect Ratio
Adult HC
Premanifest
Mild
Severe
B
Average of Nucleus Solidity
Cap Score
Average of Nucleus Aspect Ratio
Cap Score
C
Median of LaminA\C Area/Nucleus Area
Adult HC
Premanifest
Mild
Severe
Young HC
HGPS
Median of Lamin A\C Intensity
Adult HC
Premanifest
Mild
Severe
Young HC
HGPS

S7: Analysis of an additional actin cap like features

(A)Mann-Whitney's U test was performed on the medians of different actin cap like features generated from the image analysis tool mentioned above in HC fibroblast cells compared to three groups of HD (19 HC, 6 Premanifest, 10 Mild, 12 Severe), ~500 cells from each skin fibroblast sample using GraphPad 8, the results are presented in box plots using Tableau 2020.3. (B) Trend analysis was performed on the average of different actin cap like features in relation to Cap Score of different HD samples divided into three groups (Premanifest in green, Mild in blue, and Severe in red). Magenta lines are represented as least squares lines (solid line - for the average of the data, dashed lines - for the maximum and the minimum of the data) Magenta points are an average point of each group presented in a plot using MATLAB R2021a.(C) Mann-Whitney's U test was performed on the medians of Lamin A\C area divided by the nucleus area (left graph) and Lamin A\C intensity (right graph) all the data was normalized and to 7 Adult HC, 110 wells compared to 3 HD sub-groups: (4 Premanifest-40 wells, 4 Mild-40 wells, 4 Severe-50 wells) also compared to 3 HGPS-36 wells and 2 Young HC-24 wells. using GraphPad 8, the results are presented in box plots using Tableau 2020.3.

[illegible]
